# FBXL3 serves as a suppressor of regenerative myogenesis

**DOI:** 10.3389/fimmu.2025.1575712

**Published:** 2025-07-18

**Authors:** Wei He, Shiyuan Han, Yanming Wu, Min Chen, Ting Xue, Hua You, Ying Chang, Song-Bai Liu, Yi Sun, Yinjiang Tang, Xinghong Shi, Xingyu Han, Zixin Ma, Panting Qian, Sha Geng, Chaofan Wu, Yating Liang, Yangxin Li, Yan Xu, Yao-Hua Song

**Affiliations:** ^1^ Cyrus Tang Medical Institute, Collaborative Innovation Center of Hematology, State Key Laboratory of Radiation Medicine and Protection, Soochow University, Suzhou, China; ^2^ Department of Medical Laboratory, The Second Affiliated Hospital of Hainan Medical University, Haikou, Hainan, China; ^3^ Department of Cardiology, Suzhou Ninth People’s Hospital, Suzhou Ninth Hospital Affiliated to Soochow University, Suzhou, China; ^4^ Suzhou Key Laboratory of Medical Biotechnology, Suzhou Vocational Health College, Suzhou, China; ^5^ Department of Pulmonary Vascular and General Medicine, Fuwai Yunnan Cardiovascular Hospital, Yunnan Provincial Cardiovascular Disease Clinical Medical Center/Affiliated Cardiovascular Hospital of Kunming Medical University, Kunming, Yunnan, China; ^6^ Department of Cardiovascular Surgery of the First Affiliated Hospital & Institute for Cardiovascular Science, Soochow University, Suzhou, Jiangsu, China; ^7^ Department of General Medicine, The Second Xiangya Hospital, Central South University, Changsha, Hunan, China

**Keywords:** satellite cell, muscle, regeneration, FBXL3, differentiation

## Abstract

Muscle regeneration hinges on the proliferation and differentiation of satellite cells. FBXL3, a member of the F-box protein family known for its role as a negative regulator of the circadian clock, is implicated in myogenesis. In this study, we demonstrate the expression of FBXL3 in satellite cells of adult mice, where it acts as a negative regulator of myogenic regeneration. This regulation occurs through the promotion of ubiquitination and degradation of TCF12, a transcription factor crucial for differentiation. Loss of FBXL3 activates MyoD and myogenin, thereby augmenting myogenic differentiation and regeneration. The role of FBXL3 in muscle regeneration was also confirmed using the tamoxifen-inducible Pax7-CreER recombination system. To unravel the regulatory mechanism of MyoD and myogenin by FBXL3, we conducted RNA sequencing on *Fbxl3^+/+^* and *Fbxl3^-/-^* primary myoblasts. Gene set enrichment analysis (GSEA) revealed that FBXL3 deficiency enriches the gene set associated with striated muscle cell development, including MEF2C, a regulator of myogenin expression. Through a search in the ChEA3 database, TCF12 emerged as the downstream candidate gene regulated by FBXL3 to modulate MEF2C. ChIP-PCR assays confirmed the enrichment of TCF12 on MEF2C promoter at three consensus sites. Dual-luciferase reporter assay validated that TCF12 activates the MEF2C promoter. This comprehensive study underscores the crucial role of FBXL3 in satellite cell-mediated myogenic regeneration and provides insights into the intricate regulatory network involving TCF12 and MEF2C.

## Graphical Abstract

FBXL3 plays a pivotal role in regulating the proliferation and differentiation of satellite cells, thereby facilitating regenerative myogenesis. The SCF (SKP1-CUL1-F-box) complex, consisting of Skp1, Cullin1, Rbx1, and the F-box protein, is integral to this process. The F-box protein, with its COOH terminal, specifically recognizes various substrate proteins, including the transcription factor protein TCF12. FBXL3, a member of the F-box protein family, encodes the F-box domain and serves as one of the four subunits in the SCF complex. Deletion of FBXL3 in the satellite cells of mice leads to a reduction in the degradation of TCF12. Consequently, there is an accumulation of TCF12, which activates the MEF2C promoter, ultimately promoting myogenic proliferation and differentiation.

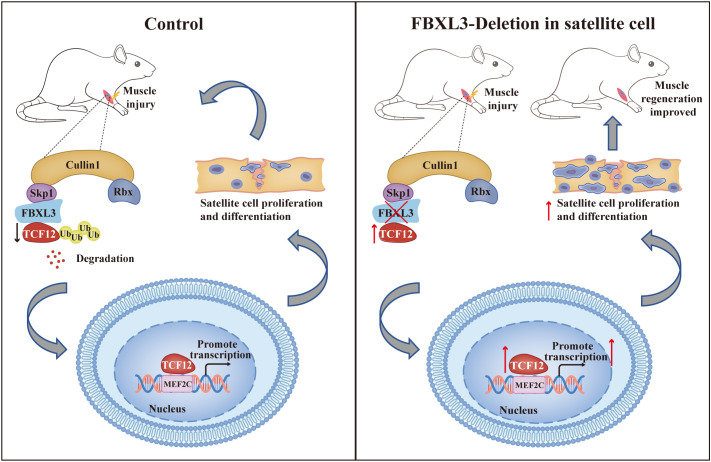

## Introduction

Skeletal muscle, as a highly adaptable organ, houses resting satellite cells positioned between the plasma membrane and the basal lamina. These local stem cells play a crucial role in the growth, maintenance, and repair of damaged muscle fibers (1–3). In response to muscle injury, myogenesis is triggered, promoting satellite cells to undergo differentiation and ultimately fuse to create myotubes (4–6). The onset of myogenic differentiation occurs as myoblasts exit the cell cycle. This is succeeded by the upregulation of myogenic regulatory factors (MRF), initiating the expression of muscle-specific genes. This process ultimately culminates in myoblast fusion, giving rise to the formation of new myofibers. MRFs, classified as basic helix-loop-helix (bHLH) transcription factors, encompass Myf5, MyoD, myogenin, and MRF4. The regulation of MRFs involves TCF12, which aids in myogenic differentiation by interacting with the E-box regions of MyoD and myogenin (7). Additionally, Myocyte enhancer factor 2C (MEF2C) influences the transcription of myogenin (8). Yet, the connection between TCF12 and MEF2C remains elusive.

Emerging evidence suggests that satellite cell function is under the control of circadian clock (9, 10). Two key components of this system are the proteins BMAL1 and CLOCK. BMAL1 and CLOCK form a heterodimer that binds to E-box elements in the promoter regions of target genes, including Period (Per) and Cryptochrome (Cry). The PER and CRY proteins subsequently form complexes that inhibit the activity of the BMAL1-CLOCK complex, creating a negative feedback loop. In addition to BMAL1 and CLOCK, there are other regulatory proteins and feedback loops involved in the circadian clock, creating a complex and finely tuned system that coordinates biological processes with the external environment.

F-box and leucine-rich repeat protein 3 (FBXL3) belong to the FBXL family and encode a protein featuring both an F-box and a leucine-rich repeat (LRR) motif. The F-box component has the ability to bind to S-phase kinase-associated protein 1 (SKP1), facilitating the formation of the SKP1-CUL1-F-box (SCF) complex by combining with Cullin 1 (CUL1) and Ring box 1 (RBX1) (11). The SCF-FBXL3 complex possesses E3 ubiquitin ligase activity, promoting the ubiquitination and subsequent degradation of CRY1 and CRY2 (cryptocrhome 1 and 2), thereby contributing to mammalian circadian rhythm (12). FBXL21, a homolog of FBXL3, acts in opposition to FBXL3 by stabilizing CRY proteins and sequestering them from FBXL3, thereby counteracting FBXL3’s activity (13, 14). *Psttm* mice, expressing a hypomorphic FBXL21 mutant, display impaired muscle differentiation and function (15, 16). Additionally, FBXL3 plays a role in the proliferation and migration of non-small cell lung cancer cells (17). Nevertheless, the precise role of FBXL3 in satellite cell function and skeletal muscle regeneration remains unclear.

This research demonstrated that FBXL3 serves as a suppressor of skeletal muscle differentiation. Targeted deletion of FBXL3 in satellite cells enhances myogenic regeneration. FBXL3 acts as an E3 ligase, coordinating the breakdown of TCF12 via the proteasome. When FBXL3 is lacking, the breakdown of TCF12 are hindered, causing an accumulation of TCF12. This accumulation triggers the activation of the MEF2C promoter, thereby promoting myogenic proliferation and differentiation.

## Materials and methods

### Animals

C57BL/6J (Wild Type) mice were purchased from Shanghai Slake Laboratory Animal Co. LTD. *Fbxl3^scko^* mice were generated by crossing Pax7-Cre (Pax7tm1 (cre) Mrc/J, The Jackson Laboratory, stock# 010530) mice with *Fbxl3^fl/fl^* mice (B6;129- *Fbxl3^tm1Nju^*, Model Animal Research Center of Nanjing University). *Fbxl3^fl/fl-Pax7cre-ERT2^* mice were generated by crossing Pax7-CreERT2 (Pax7tm1 (cre) Gaka/J, The Jackson Laboratory, stock# 017763) mice with *Fbxl3^fl/fl^* mice. For deletion of *Fbxl3*, *Fbxl3^fl/fl-Pax7cre-ERT2^* mice were intraperitoneally injected with tamoxifen (80 mg/kg;MCE, HY-13757A) dissolved in corn oil (MCE, HY-Y1888) for 5 consecutive days. The genotype of mice was identified by PCR using genomic DNA and agarose gel electrophoresis. The sequence of the PCR primers is shown in **Supplementary Table S1**. RNA and protein were extracted from satellite cells of *Fbxl3^fl/fl^* and *Fbxl3^scko^* mice, and relative mRNA and protein expression of FBXL3 was analyzed by RT-qPCR and Western blot, respectively. The RT-qPCR primers are described in **Supplementary Table S1**. All mice used in this study were males and maintained in a Special Pathogen Free animal facility at Soochow University. Mice were kept in individually ventilated cages (IVC), Euro Standard Type IIL in groups of six with filter tops, and provided access to drinking water and standard chow ad libitum. The temperature in animal facilities was 22–26°C, and the humidity was 55 ± 10%. The light cycle was under 14 hr-light and ten hr-dark cycles. All animal experiments comply with the ARRIVE guidelines and are carried out in accordance with the National Institutes of Health guide for the care and use of laboratory animals (NIH Publications No. 8023, revised 1978), and the manuscript has followed such guidelines. All animal protocols were approved by the Institutional Laboratory Animal Care and Use Committee of Soochow University.

### Ischemia-reperfusion injury model

The hip joint of *Fbxl3^fl/fl^* and *Fbxl3^scko^* mice was placed an 1/8 orthodontic rubber band (ORMAER, SH-533) for 3 h, followed by reperfusion for 24 h.

### Preparation of frozen muscle sections

The tibial anterior (TA), gastrocnemius (GA), and quadriceps (Q) were removed, embedded in OCT (Leica, 14020108926), then frozen in isopentane cooled in liquid nitrogen. Ten μm thick transverse sections were prepared in a microtome cryostat (Leica CM 1950, Weztlar, Germany).

### Histological and morphometric analysis

For the assessment of muscle morphology, the sections were stained with Hematoxylin and Eosin kit (Beyotime, C0105). Sections were treated as follows: deionized water for 5 min x 3, Hematoxylin for 5 min, tap water for 5 min x 3, deionized water for 1 min, Eosin for 2 min, 95% ethanol for 30 s, 100% ethanol for 1 min, and xylene for 5 min.

### Isolation and culture of single myofibers

Single myofibers were isolated from the extensor digitorum longus (EDL) muscle of 6–8 weeks old male *Fbxl3^fl/fl^* or *Fbxl3^scko^* and enzymatically digested in DMEM supplemented with 2 mg/mL collagenase type I (Rockland, MB-118-0100) at 37°C for 2 h. Digestion was terminated with preheated DMEM medium containing 10% fetal bovine serum (FBS, EallBio, 03.C16001DC). The fibers were gently detached from the muscle using a Pasteur pipette. The single myofibers were transferred to preheated DMEM without serum and cultured at 37°C with 5% CO_2_ for 1 h. The single myofibers were plated on 10% matrigel (Corning, 5089301) coated dishes and cultured with myoblasts growth medium consisting of DMEM with 20% FBS, and 10 ng/mL basic fibroblast growth factor at 37°C with 5% CO_2_ for three days. The cultured fibers were fixed by using a fixative solution (Beyotime, P0098) for immunofluorescence staining.

### Isolation and culture of satellite cells

Satellite cell-derived primary myoblasts were isolated from the TA muscle of 8 weeks old male *Fbxl3^fl/fl^* and *Fbxl3^scko^* mice or WT mice (18). TA muscle was minced and digested in DMEM (HyClone, SH30243.01) containing 2.5 mg/mL Dispase type II (Roche, 4942078001) and 10 mg/mL Collagenase D (Roche, 11088866001) for 90 min. Digestion was terminated with DMEM medium containing 10% FBS, and the digested material was filtered through a 70 μm nylon mesh (Fisher Scientific, 22363548). Cell pellets were resuspended in PBS containing 2% Fetal Bovine Serum, 2% Penicillin-Streptomycin and purified by FACS sorting using the following antibodies: FITC-conjugated anti-CD34 (Thermo Fisher Scientific, 11-0341-81, 1:100 dilution), Alexa647-conjugated anti-α7-Integrin (R&D systems, FAB3518R-100UG, 1:100 dilution) and PE-conjugated anti-CD31 (Biolegend, 102408, 1:100 dilution), anti-CD45 (BD Biosciences, 561087, 1:100 dilution), anti-Sca-1 (BD Biosciences, 553108, 1:100 dilution), and anti-CD11b (BD Biosciences, 557397, 1:100 dilution). Unbound antibodies were washed with PBS, and the suspension was filtered by the 70 µm nylon mesh again. Cell doublets were removed based on forward scatter and side scatter profiles. The cells were immunostained with PE-labeled CD11b, Sca-1, CD45, CD31 antibodies to remove lineage cells, followed by a tandem positive selection using Alexa647-labeled α7- Integrin and FITC-labeled CD34 antibodies. Flow cytometry was performed using a Flow Cytometer (FACSAria III). Finally, the cells were cultured in the myoblast growth medium (Shanxi Anning Yunsheng Biotechnology Co., LTD, 60071-1) on dishes coated with rat tail collagen (Gibco, A1048301).

### Myogenic differentiation

Primary myoblasts/C2C12 were seeded at 5 x 10^5^ cells/well in 6-well cell culture plates coated with 2% rat tail collagen. After 24 h, when the cell density reached 70–80% confluency, the growth medium was changed to a differentiation medium (DMEM + 2% horse serum, Gibco, 16050122). Samples were collected on day 6 for immunofluorescence staining of the myotube.

### Immunofluorescence

Frozen muscle sections, single myofibers and myoblasts were fixed in immunostaining fixative solution for 10 min (Beyotime, P0098), permeabilized in 0.2% Triton X-100 (Sigma-Aldrich, 9002-93-1) for 10 min, and then blocked in immunostaining blocking solution (Beyotime, P0102) for 1 h at room temperature. The muscle sections or myoblasts were incubated with primary antibodies in a blocking solution at 4°C overnight. After three washes in PBS, secondary antibodies were added and incubated for 1 h at room temperature. Anti-FBXL3 was obtained from OriGene (AP26069PU-N, 1:100), antibodies against Pax7 (clone Pax7-s, 1:10 dilution), eMyHC (clone F1.652-s, 1:100 dilution), MyHC (clone MF20-s, 1:100 dilution) and Myogenin (clone F5D-s, 1:10 dilution) were from DSHB. Anti-laminin antibody was from Sigma-Aldrich (L9393, 1:500, dilution). The MyoD antibody (sc-377460, 1:100 dilution), TCF12 antibody (sc-28364, 1:100 dilution) and dystrophin antibody (sc-58754, 1:150 dilution) was from Santa Cruz Biotechnology. The secondary antibodies were purchased from Thermo Fisher Scientific, which include Alexa Fluor 488 goat anti-mouse IgG1 (A21121, 1:500 dilution), Alexa Fluor 555 goat anti-mouse IgG2b (A21147, 1:500 dilution), Alexa Fluor 568 goat anti-rabbit IgG (H + L) (A11011, 1:500 dilution), Alexa Fluor 647 goat anti-mouse IgG1 (A21240, 1:500 dilution). Finally, nuclei were counterstained with DAPI (Sigma, D8417, 1:1000 dilution). The images were taken by confocal microscope (Olympus Fluoview FV3000, Olympus) and analyzed with Olympus FV31S-SW software.

### Quantification of imaging data

The total number of centronucleated fibers (CNF), or myofibers containing more than one central nucleus in TA muscle sections, was performed using the Point Tool Counter of ImageJ (NIH). The average CSA of regenerating myofibers was analyzed using ImageJ software installed with the Open-CSAM macro (19).

For the single myofiber experiment, analysis was performed on 10–15 myofibers for each mouse at each time point, and 3 mice per mouse strain were analyzed. Quantification of Pax7^+^ and MyoD^+^ cells in muscle sections was performed manually using the Point Tool Counter of ImageJ (NIH) by an investigator who is blinded to the research design. The differentiation index (percentage of nuclei within MyHC^+^ myotubes) was calculated by counting 1,200 cells (n = 3).

Fluorescence co-localization analysis was performed using ImageJ software.

### RNA isolation and RT-qPCR assay

RNA was extracted using the RNA simple Total RNA Kit (Tiangen, DP419) according to the manufacturer’s instructions. Total RNA (1 μg) was reverse transcribed into cDNA using ReverAidTM First Strand cDNA Synthesis (Thermo Fisher Scientific, k1622). Real-time quantitative PCR (RT-qPCR) was performed using SYBR Premix Ex Taq (Takara, RR420B) and the QuantStudio 6 Flex Real-Time PCR System (Thermo Fisher Scientific). Relative expression was calculated from cycle threshold (Ct; relative expression = 2^–(S△Ct – C△Ct)^) values using *Gapdh* as the internal control for each sample. The sequences of RT-qPCR primers are shown in **Supplementary Table S1**.

### RNA sequencing and bioinformatic analysis

RNA-seq was performed by Shanghai Biotechnology Corporation (SBC). Satellite cells were isolated from the hind limbs of 8 weeks old male *Fbxl3^fl/fl^* and *Fbxl3^scko^* mice. Total RNA was extracted using the RNAsimple total RNA Kit per the manufacturer’s instructions. The quality of the total RNA was inspected by Agilent Bioanalyzer 2100 (Agilent Technologies, Santa Clara, CA, US). Qualified total RNA was further purified by RNAClean XP Kit (Beckman Coulter, A63987). The cDNA library was generated using a VAHTS Universal V6 RNA-seq Library Prep Kit for Illumina (Vazyme, NR604-02) and sequenced on an Illumina system (Illumina). Gene set enrichment analysis (GSEA) was performed using GSEA version 4.3.1 software. ChEA3 datebase (20) was used to analyze the transcriptional factors of the leading edge subset genes involved in muscle cell differentiation downstream of FBXL3. The potential binding sites of TCF12 to MEF2C promoter were analyzed using JASPAR prediction (21) (http://jaspar.genereg.net/).

### Western blot

Cells or muscles were lysed in lysis buffer (Cell Signaling Technology, 46232S) in the presence of protease inhibitor PMSF (Amresco, BY12203) and phosphatase inhibitors (Roche, PhosSTOP EASYpack, 5892970001) for 30 min on ice. The lysates were centrifuged at 13,000g (Thermo Fisher Scientific, MicroCL21R, 41282794) for 30 min to remove undissolved particles. Equal amounts of protein extracts were separated by SDS-PAGE and blotted to PVDF membranes (Millipore, ISEQ00010). The membranes were blocked by 5% non-fat dry milk dissolved in 0.1% TBS-Tween (Beyotime, ST825) for 2 h at room temperature, followed by incubation with primary antibodies overnight at 4°C. FBXL3 antibody was from OriGene (AP26069PU-N, 1:1000 dilution). MyoD (sc-377460, 1:1000 dilution) and TCF12 antibody (sc-28364, 1:500 dilution) were from Santa Cruz Biotechnology. Antibodies against Myogenin (clone F5D-s, 1:500 dilution) were from DSHB. Antibodies against GAPDH (2118s, 1:5000 dilution) were from Cell Signaling Technology. Antibodies against CRY2 (SAB1300079, 1:1000) and Flag (F7425, 1:800) were purchased from Sigma. After three washes in 0.1% TBS-Tween, membranes were incubated for 1h with HRP-conjugated secondary antibodies (Cell Signaling Technology, anti-mouse IgG, 7076S, 1:3000 dilutions; anti-rabbit IgG, 7074S, 1:5000 dilutions). After washing, the membranes were incubated with an enhanced chemiluminescence reagent (PerkinElmer, NEL120001EA). Western blot stripping was performed using the stripping buffer (Beyotime, P0025B).

### Construction of lentivirus overexpressing Fbxl3/Tcf12

*Fbxl3* cDNA was cloned into the LV5 plasmid (Puromycin resistance, GenePharma) with SphI and BamHI restriction enzymes. Tcf12 cDNA was cloned into the LV5 plasmid with SphI and NotI restriction enzymes. The 293T cells were seeded at 1 x 10^7^ cells in a 100 mm dish with 10% FBS in DMEM. As the cell density approaches 60% confluency, the recombinant LV5 plasmid (10 μg) and packaging plasmids △R8.74 (6.5 μg), VSV-G (3.5 μg) and Rev (2.5 μg) were co-transfected into 293T cells to produce lentiviral particles. After 48h of culture, viral supernatant was collected and filtered through a 0.22μm syringe filter. Myoblasts were seeded at 1 x 10^5^ cells/well in a 6-well cell culture plate. When the cell density reached 50% confluency, the virus supernatant and culture medium were added in a 1:1 ratio. After transfection for 72 h, puromycin (5 μg/mL) was added, and the selection was continued for five days. The packaging plasmids were provided by Dr. Yun Zhao, Soochow University. The sequences of *Fbxl3* and *Tcf12* PCR primers are shown in listed in **Supplementary Table S1**.

### 293T cell culture and transfection

The 293T cells were cultured in DMEM containing 10% FBS. For immunoprecipitations, 2 × 10^6^ cells were plated into 60 mm dishes 1 day before transfection, and plasmids were transfected into the cells using Lipofectamine (2000) (Thermo Fisher Scientific, 11668019). TCF12 degradation assays were performed by transfecting TCF12 plasmid into 2 × 10^5^ 293T cells in the presence or absence of FBXL3 plasmid in a 12-well plate. Thirty-six hours after transfection, 100 μg/ml cycloheximide (CHX, MedChemExpress, HY-12320) was added and cells were collected at the indicated times. MG132 (HY-13259) were purchased from MedChemExpress.

### Co-immunoprecipitation

The Co-IP assay was carried out using the IP/Co-IP Kit (Absin, abs955). Ten million cells were centrifuged at 300g for 5 minutes. After washing with pre-cooled PBS solution twice, the cell pellet was lysed in lysis buffer containing PMSF (Lysis buffer: PMSF=100:1) on ice for 30 minutes. After centrifugation, the supernatant was transferred to a new tube, and the protein concentration was determined by BCA protein quantification kit. The supernatant was diluted to 2 mg/ml, 50 μl of total protein solution was taken as input, and another 500 μl of protein was taken as IP sample. Pre-clearing step by adding 5 μl of Protein A and 5 μl of Protein G agarose beads to the protein sample to reduce non-specific binding, the mixture was incubated at 4°C for 2 hours and subsequently centrifugation at 2,400g for 1 minute at 4°C, the supernatant was transferred to a new tube to incubate with the primary antibody anti-TCF12 antibody or anti-FBXL3 antibody (4μl per 1 x 10^7^ cells) overnight at 4°C. The next day, 5 μl of Protein A and G agarose beads was added and incubated at 4°C for 4 hours. After centrifugation at 14,000g at 4°C for 1 minute, the agarose beads were washed 3 times with wash buffer, and 30 μl of SDS buffer was added to precipitate. Finally, after adding 50 μl of 2 x Loading buffer to the input, the IP samples and input were placed into a 95°C metal bath for 5 minutes to denature the protein. Afterward, supernatants were subjected to WB analysis.

### Chromatin immunoprecipitation

The ChIP assay was carried out using the Chip-IT Express Kit (Active motif, 53008). Myoblasts were cross-linked with 1% formaldehyde for 10 min at room temperature and then incubated on ice for 30 min with lysis buffer. The nuclei were separated by centrifugation at 4°C 2,400g for 10 min and then resuspended with shearing buffer. The DNA samples were sonicated to fragment sizes of 200–500 bp. After pre-clearing with normal IgG, the anti-TCF12 antibody (4μl per 1 x 10^7^ cells) and protein G Magnetic beads were added and incubated at 4°C overnight. After washing, the cross-link was reversed, and DNA was purified. PCR primers were designed every 250 bp, covering the entire MEF2C promoter region 2000 bp upstream of the transcription start site. The immunoprecipitated DNA was amplified by PCR. Quantification of fold enrichment was performed by RT-qPCR, and the data were expressed as the percentage enrichment of input. The enrichment of MEF2C was detected using specific primers described in **Supplementary Table S1**.

### Dual luciferase reporter assay

MEF2C-Luciferase (MEF2C-Luc) plasmid was constructed by ligating the MEF2C promoter to the PGL4.17 plasmid respectively. MEF2C-Luc along with an empty vector or TCF12 overexpression plasmid (TCF12-cDNA), and a pRL-TK plasmid Renilla luciferase control reporter (RL-TK) plasmid was transfected into 293T by Lipofectamine (2000) (Thermo Fisher Scientific, 11668019). The dual-luciferase reporter assay was performed 72 h after transfection using the Dual-Luciferase Reporter Assay System (Promega, E1910), and the signal was detected by a luminometer (Luminoskan Ascent, Thermo Fisher Scientific) (22).

### Circadian gene expression in TA muscles

*Fbxl3^fl/fl^* and *Fbxl3^scko^* male mice (6–8 weeks old) were entrained at 12-h light and 12-h dark cycles (8 a.m.–8 p.m. light and 8 p.m. to 8 a.m. dark) for two weeks prior to the experiments. Zeitgeber time 0 (ZT0) and ZT12 correspond to 8 a.m. and 8 p.m, respectively. TA muscles were isolated every 4 h starting at ZT2 (23). Total RNA was extracted from TA muscles, and the mRNA levels of circadian clock-related genes in TA muscle of *Fbxl3^fl/fl^* and *Fbxl3^scko^* mice were analyzed by RT-qPCR. The sequences of the primers are shown in **Supplementary Table S1**.

### Grip strength measurement

Mouse grip strength was measured using a Grip Strength Meter (YLS-13A, Ji-Nan Biotechnology). The mice were acclimated to the meter for 10 min before the test. The mouse was allowed to grasp the metal pull bar with the forepaws or with all four paws. Grip strength of the forelimbs or four - limbs was measured by allowing the mice to grasp the metal lever with their front paws or all four paws, respectively (22). The mouse was gently pulled backward in the horizontal plane by holding the tail until it could no longer grasp the bar. Peak tension was recorded at the time of release. Five tests were performed for each mouse with a 40-s break between tests. Forelimb or total grip strength was defined as the average peak tension from the three best attempts (24).

### Statistics

All datasets were tested for normality with the Shapiro-Wilk test and the homogeneity of the variances with the F test (two groups) or Brown-Forsythe test (more than two groups), followed by either parametric or nonparametric statistical analysis. Parametric tests were used to analyze variables with equal variance and normal distribution, whereas non-parametric tests were used to analyze variables without normal distribution. All analyses were performed using GraphPad Prism 8.0 software, and the sample size, as well as details of statistical analysis, are indicated in the figure legends.

The unpaired Student’s t test was used to compare two groups when the values were normal distribution and equal variance. If the variance was unequal, the unpaired t test with Welch’s correction was used. If the data failed the normality test, the Mann-Whitney Wilcoxon test was used. One-way analysis of variance (ANOVA) with *post-hoc* Tukey’s multiple comparisons was used to analyze the comparison among more than two groups when the values are normal distribution and equal variance. If the variance was unequal, the Brown-Forsythe and Welch ANOVA tests with *post-hoc* Dunnett’s T3 multiple comparisons were used. If the data failed the normality test, a Kruskal-Wallis test with Dunn’s multiple comparisons was run to compare the groups. The Data are expressed as means ± standard deviation (SD), and the difference of *P* < 0.05 was considered statistically significant.

## Results

### FBXL3 is present in satellite cells of adult mice

**Figure 1:**
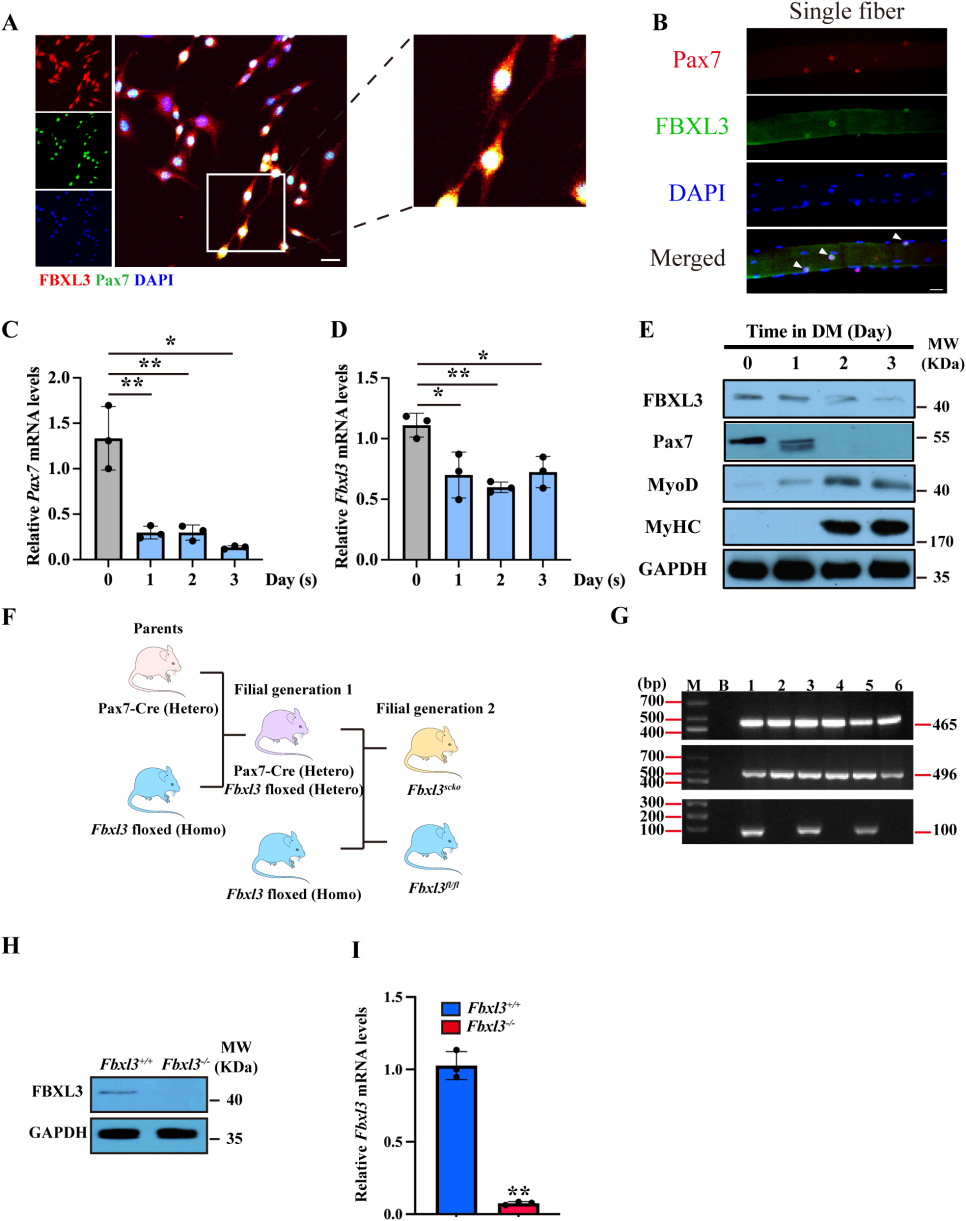
FBXL3 is expressed in satellite cells and negatively correlated with differentiation. **(A)** Primary myoblasts isolated from the TA muscle of C57BL/6J (WT) mice were immunostained with antibodies against FBXL3 (red) and PAX7 (green). The nuclei were stained with DAPI (blue). Cells cultured in growth medium for 48 hours. Scale bars: 20 μm. **(B)** Single myofibers were isolated from the EDL muscle of C57BL/6J (WT) mice and immunostained for Pax7 (red) or FBXL3 (green). Nuclei were identified by staining with DAPI (blue). Scale bar: 20 μm. **(C)** RT-qPCR analysis of the expression levels of *Pax7* in myoblasts cultured in a differentiation medium for three days (n=3). *P* values were determined by unpaired t test. **(D)** RT-qPCR analysis of the expression levels of *Fbxl3* in myoblasts cultured in a differentiation medium for three days (n=3). *P* values were determined by unpaired t test. **(E)** Western blot analysis of the levels of FBXL3, Pax7, MyoD and MyHC in primary myogenic cells cultured in differentiation medium (DM) containing 2% horse serum. GAPDH was used as loading control. **(F)** Breeding strategy for obtaining *Fbxl3^scko^* and littermate *Fbxl3^fl/fl^* mice. **(G)** The genotype of *Fbxl3^scko^* and littermate *Fbxl3^fl/fl^* mice were identified by PCR and agarose gel electrophoresis using genomic DNA. FBXL3-floxed (Homo) mice genotype PCR product is 465 bp. For the Pax7-cre mice, wild type genotype PCR product is 496 bp, mutant PCR product is 100 bp, and heterozygote PCR product is 496 and 100 bp. Mouse #1, 3, 5 had 465 bp, 496 bp and 100 bp bands; therefore, these mice are *Fbxl3^scko^*. Mouse #2, 4, 6 had 465 and 496 band; therefore, these mice are FBXL3-floxed (Homo). M: marker, B: blank, no template DNA control. **(H)** Western blot analysis of the protein level of FBXL3 in primary myoblasts of *Fbxl3^fl/fl^* and *Fbxl3^scko^* mice. **(I)** RT-qPCR analysis of mRNA levels of *Fbxl3* in primary myoblasts from *Fbxl3^fl/fl^* and *Fbxl3^scko^* mice (n=3). *P* values were determined by unpaired t test with Welch’s correction. Values are mean ± SD. *P < 0.05, **P < 0.01.

To confirm the expression of FBXL3 in satellite cells, we isolated primary myoblasts and single myofibers from extensor digitorum longus (EDL) muscle from 8-week-old wild-type mice and conducted immunostaining for FBXL3 and PAX7. Confocal microscopy of myoblasts revealed that PAX7 is localized in the nucleus, while FBXL3 is expressed in both the cytoplasm and nucleus of the same cells (**Figures 1A, B**, **Supplementary Figures S1A, B**). To explore the correlation between PAX7 and FBXL3, we extracted RNA and protein from primary myoblasts at various time points during differentiation. RT-qPCR and Western blot results indicated that both *Pax7* and *Fbxl3* levels decrease during differentiation (**Figures 1C–E**), suggesting that the downregulation of FBXL3 may be necessary for myogenic differentiation.

To investigate the function of FBXL3 in myogenesis, we generated mice with satellite cell-specific FBXL3 knockout (*Fbxl3^scko^*) by breeding *Fbxl3^fl/fl^* mice with PAX7-Cre mice (**Figure 1F**), and the genotype was confirmed using PCR (**Figure 1G**). Primary myoblasts from the TA muscle of *Fbxl3^fl/fl^ and Fbxl3^scko^* mice were isolated through FACS sorting (**Supplementary Figure S1A**). The efficacy of FBXL3 deletion in satellite cells was validated through Western blot (**Figure 1H**), RT-qPCR (**Figure 1I**), and immunostaining of FBXL3 and PAX7 in *Fbxl3^+/+^* and *Fbxl3^-/-^* primary myoblasts (**Supplementary Figure S1C**).

FBXL3, an F-box protein that is responsible for the ubiquitination and degradation of cryptochromes (Crys) (11), prompted an analysis of Cry2 expression in *Fbxl3^+/+^*, *Fbxl3^-/-^* primary myoblasts, and other PE^+^ lineage cells isolated by Flow Sorting. The results revealed a significant increase in Cry2 protein expression in *Fbxl3^-/-^* cells compared to *Fbxl3^+/+^* and PE^+^ cells (**Supplementary Figures S1D–F**). The purity of primary myoblasts was confirmed through PAX7 immunofluorescence staining (**Supplementary Figures S1G, H**). Additionally, the expression of other clock genes was examined over a 24-hour period using RT-qPCR. The data showed a notable downregulation in gene expression levels of *Fbxl3* and a significant upregulation of both *Cry1* and *Cry2* (**Supplementary Figure S2**).

### Deletion of FBXL3 in satellite cells enhances the regeneration of injured myofibers

**Figure 2:**
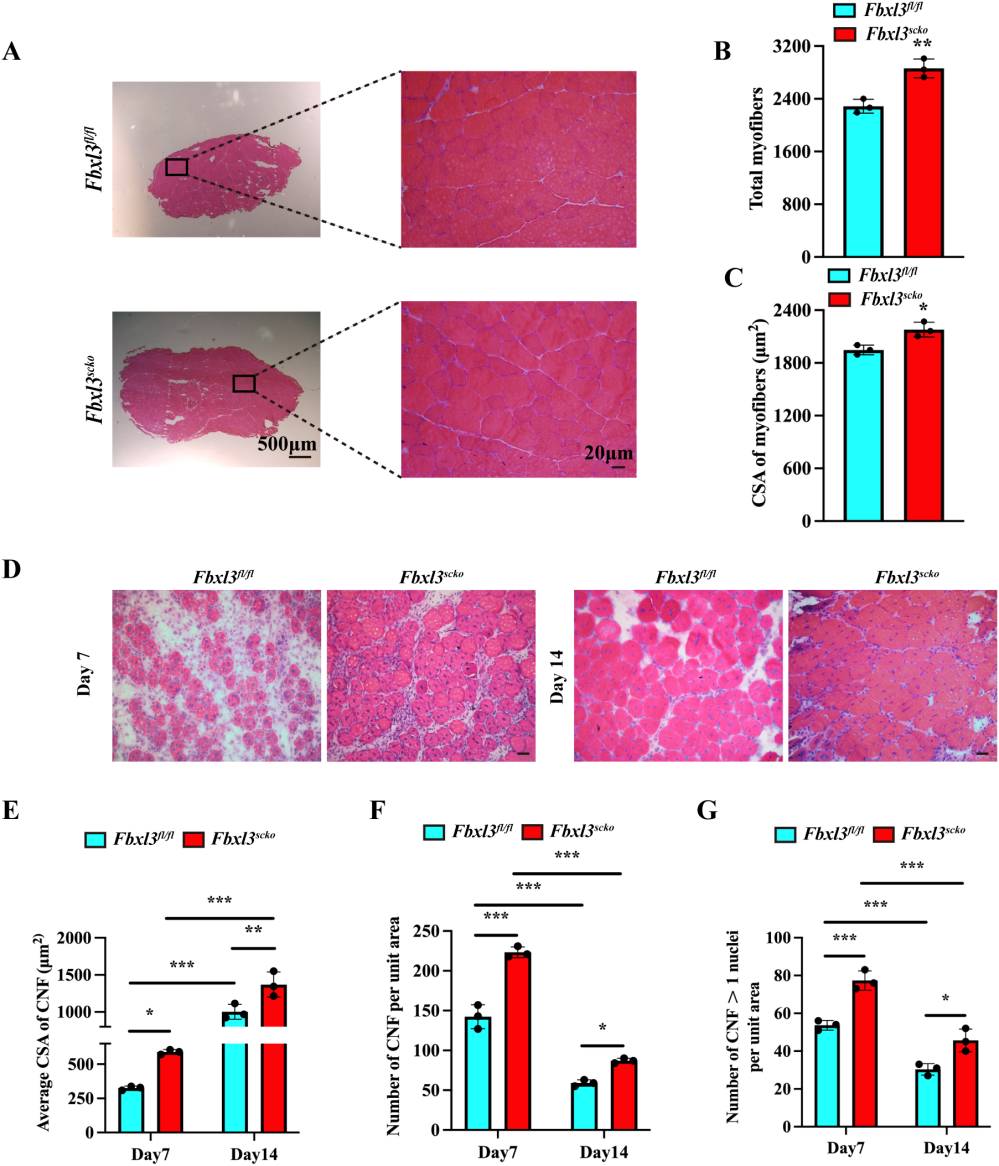
Loss of FBXL3 enhances muscle repair after IR injury. **(A)** H&E staining of uninjured TA muscles from *Fbxl3^scko^* and *Fbxl3^fl/fl^* littermates. **(B)** Quantification of the number of total myofibers in uninjured TA muscle sections, *P* values were determined by unpaired t test. **(C)** The average cross-sectional area (CSA) of uninjured TA muscle myofibers, *P* values were determined by unpaired t test. **(D)** H&E-stained sections of the TA muscle from *Fbxl3^scko^* and *Fbxl3^fl/fl^* littermates at indicated time points after IR injury. Scale bars: 20 μm. **(E-G)** Quantification of myogenic regeneration. Average CSA of regenerating myofibers **(E)**, numbers of centronucleated fibers (CNF) per field (0.1 mm^2^) **(F)**, and the number of myofibers containing more than one central nucleus **(G)** at 7 and 14 days after IR injury (n=3). *P* values were determined by One-way analysis of variance (ANOVA) with *post-hoc* Tukey’s multiple comparisons. Values are mean ± SD. *P < 0.05, **P < 0.01, ***P < 0.001.

Deletion of FBXL3 in satellite cells resulted in a significant increase in body size, body and muscle weight, and grip strength compared with littermate *Fbxl3^fl/fl^* mice (**Supplementary Figures S3A–F**). H&E staining revealed a significant increase in both the number and size of muscle fibers in *Fbxl3^scko^* mice compared to *Fbxl3^fl/fl^* mice (**Figures 2A–C**).

The impact of FBXL3 deletion on muscle regeneration was evaluated using the ischemia reperfusion (IR) injury model. Compared to littermate *Fbxl3^fl/fl^* mice, the tibial anterior muscle (TA) of *Fbxl3^scko^* mice exhibited higher numbers of centronucleated fibers (CNF) and a greater cross-sectional area (CSA) of CNF after IR injury (**Figures 2D–G**, **Supplementary Figure S4A**).

**Figure 3:**
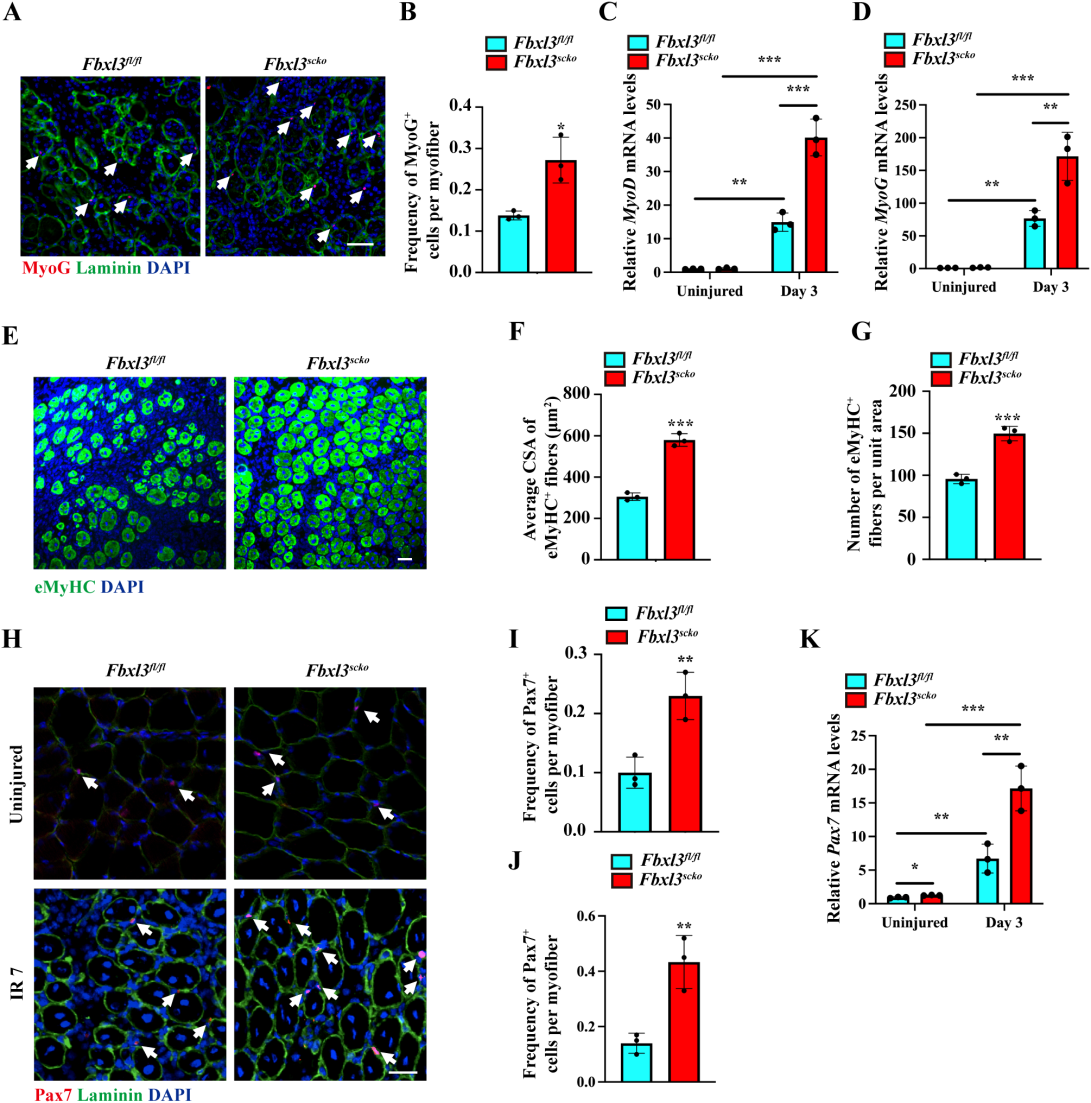
Loss of FBXL3 promotes the expression of myogenic markers after IR injury. **(A, B)** Representative immunofluorescence staining and quantification of the average number of Myogenin^+^ cells per myofiber of IR injured TA muscle from *Fbxl3^scko^* and *Fbxl3^fl/fl^* mice 7 days post-injury. The nuclei were stained with DAPI (blue). Scale bars: 100 μm. *P* values were determined by unpaired t test. **(C, D)** RT-qPCR analysis of relative mRNA levels of *MyoD* and *myogenin* in uninjured and IR-injured TA muscles of *Fbxl3^scko^* and *Fbxl3^fl/fl^* mice. *P* values were determined by One-way analysis of variance (ANOVA) with *post-hoc* Tukey’s multiple comparisons. **(E-G)** Immunostaining of eMyHC (green), quantification of average cross-sectional area (CSA) of eMyHC^+^ fibers, and the number of eMyHC^+^ fibers per field (0.08 mm^2^) in TA muscles from *Fbxl3^scko^* and *Fbxl3^fl/fl^* mice 7 days after IR-induced injury. Scale bars: 50 μm. *P* values were determined by unpaired t test. **(H)** Immunostaining with Pax7 (red) and laminin (green) showing an increased number of satellite cells (white arrows) in uninjured and IR-injured TA muscles of *Fbxl3^scko^* mice compared to *Fbxl3^fl/fl^* mice. Scale bar: 50 μm. **(I-J)** Quantification of the average number of Pax7^+^ cells per myofiber in uninjured **(I)** and IR-injured **(J)** TA muscles of *Fbxl3^fl/fl^* and *Fbxl3^scko^* mice. **(K)** RT-qPCR analysis of relative mRNA levels of *Pax7* in uninjured and IR-injured TA muscles. *P* values were determined by One-way analysis of variance (ANOVA) with *post-hoc* Tukey’s multiple comparisons. n = 3 mice per group. Values are mean ± SD. *P < 0.05, **P < 0.01, ***P < 0.001.

The expression of MyoD and myogenin is essential for myogenic differentiation and regeneration. Immunostaining revealed a higher number of Myogenin^+^ cells in muscle sections from *Fbxl3^scko^* mice compared to *Fbxl3^fl/fl^* mice (**Figures 3A, B**). RT-qPCR and Western blot analysis demonstrated increased expression of MyoD and myogenin in the IR-injured TA muscle from *Fbxl3^scko^* mice compared to *Fbxl3^fl/fl^* mice (**Figures 3C, D**, **Supplementary Figure S4B**). The expression of the embryonic/developmental isoform of MyHC (eMyHC) was higher in the TA muscle of *Fbxl3^scko^* mice than *Fbxl3^fl/fl^* mice on day seven after injury (**Figures 3E–G**, **Supplementary Figure S4C**). The increased expression of eMyHC resulting from FBXL3 deletion suggests that FBXL3 may be involved in regulating satellite cell proliferation and differentiation. Immunofluorescence staining for Pax7 was performed to determine whether the loss of FBXL3 could affect satellite cell proliferation. Immunofluorescence staining of muscle sections revealed an increased number of Pax7^+^ cells in *Fbxl3^scko^* mice vs. *Fbxl3^fl/fl^* mice upon injury induced by IR, as well as in the uninjured muscle (**Figures 3H–J**). The increased expression of Pax7 was verified by RT-qPCR (**Figure 3K**).

**Figure 4:**
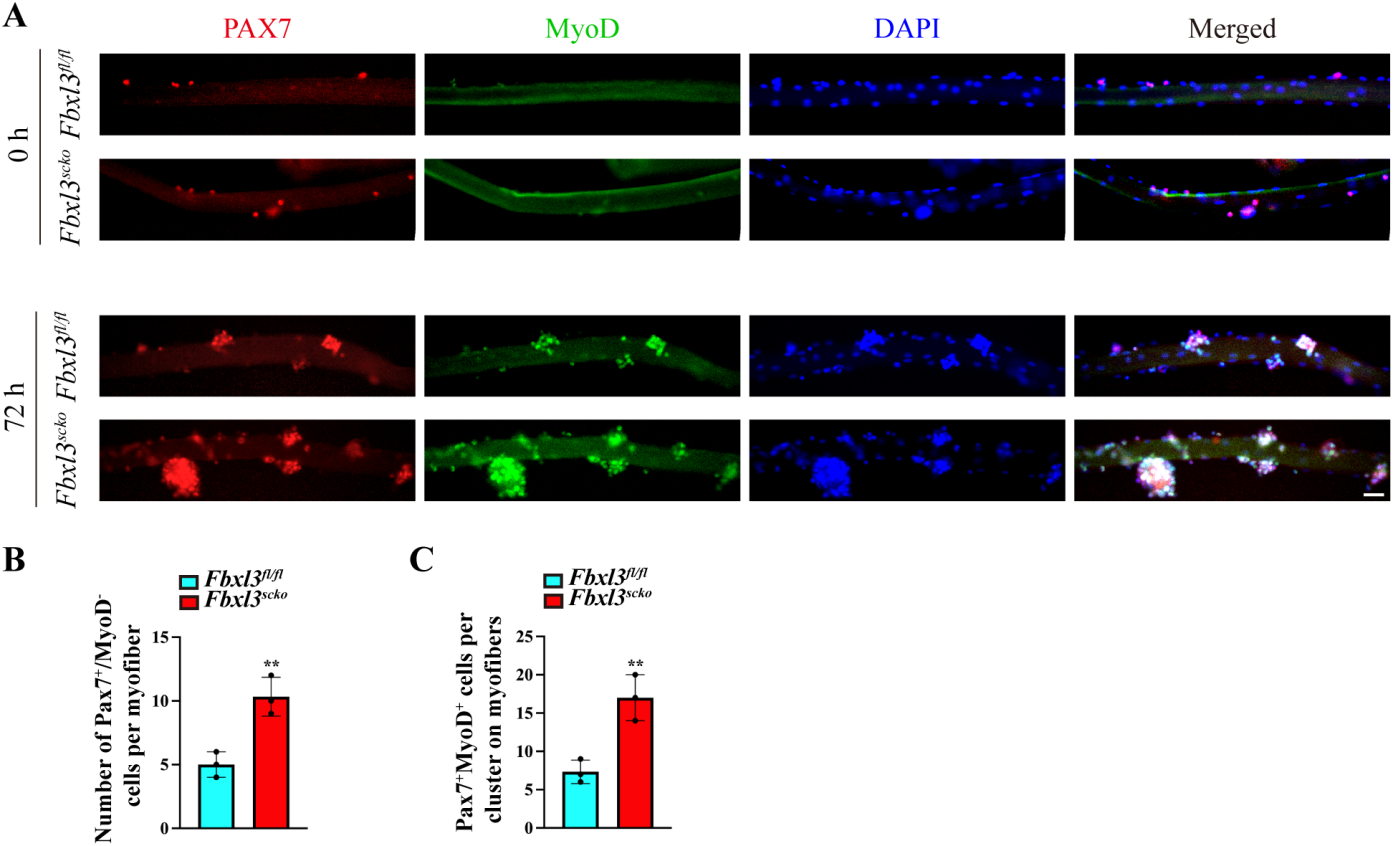
The loss of *Fbxl3* promotes the proliferation and differentiation of satellite cells. **(A)** Single myofibers were isolated from the EDL muscle of *Fbxl3^fl/fl^* and *Fbxl3^scko^* mice. The myofibers collected either immediately after isolation or after 72 h of culture were immunostained for Pax7 (red) or MyoD (green). Nuclei were identified by staining with DAPI (blue). Scale bar: 100 μm. **(B)** Quantification of the number of Pax7^+^/MyoD^−^ cells in each myofibers immediately after isolation. **(C)** Quantification of the number of Pax7^+^MyoD^+^ cells on cultured myofibers. *P* values were determined by unpaired t test. n = 3 mice per group. Values are mean ± SD. **P < 0.01.

We then isolated single myofibers from EDL muscle of *Fbxl3^fl/fl^* and *Fbxl3^scko^* mice. Myofiber-associated cells were identified by immunostaining with antibodies against Pax7 and MyoD on single myofibers. Pax7^+^/MyoD^–^ and Pax7^+^MyoD^+^ are considered as quiescent and activated satellite cells, respectively (25). Compared to the control, freshly isolated *Fbxl3^scko^* myofibers displayed an increased number of quiescent satellite cells (Pax7^+^/MyoD^–^) (**Figures 4A, B**). After 72 h in culture, these satellite cells formed clusters, and the clusters of *Fbxl3^scko^* myofibers contained more activated satellite cells (Pax7^+^MyoD^+^) compared to the control (**Figures 4A, C**).

A differentiation assay was performed utilizing *Fbxl3^+/+^* and *Fbxl3^-/-^* myoblasts isolated from the TA muscle of *Fbxl3^fl/fl^ and Fbxl3^scko^* mice via FACS sorting. Immunofluorescence staining of myosin heavy chain (MyHC) demonstrated that FBXL3 deletion enhances myogenic differentiation (**Supplementary Figures S5A, B**). Conversely, overexpression of FBXL3 in C2C12 myoblasts suppressed the expression of *MyoD* and *myogenin*, thereby inhibiting myogenic differentiation (**Supplementary Figures S5C–H**).

### FBXL3 governs satellite cell differentiation through the TCF12/MEF2C signaling pathway

To elucidate how the loss of FBXL3 promotes satellite cell differentiation, we conducted RNA sequencing and bioinformatics analysis using *Fbxl3^+/+^* and *Fbxl3^-/-^* primary myoblasts. According to the screening criteria of log_2_FC ≥ 1 or log_2_FC ≤ -1 and *P.*adj < 0.05, a total of 4006 differentially expressed genes were obtained from the sequencing results, including 2325 upregulated differentially expressed genes and 1681 downregulated differentially expressed genes (**Supplementary Figures S6A, B**). In order to identify the potential signaling pathways altered by FBXL3 deficiency, we performed Gene Ontology (GO) and Kyoto Encyclopedia of Genes and Genomes (KEGG) analysis on the sequencing results. The analysis indicated that most of the processes are related to protein-protein interactions (**Supplementary Figures S6C, D**), and we speculate that FBXL3 may regulate the function of satellite cells by influencing other related factors at the protein level.

**Figure 5:**
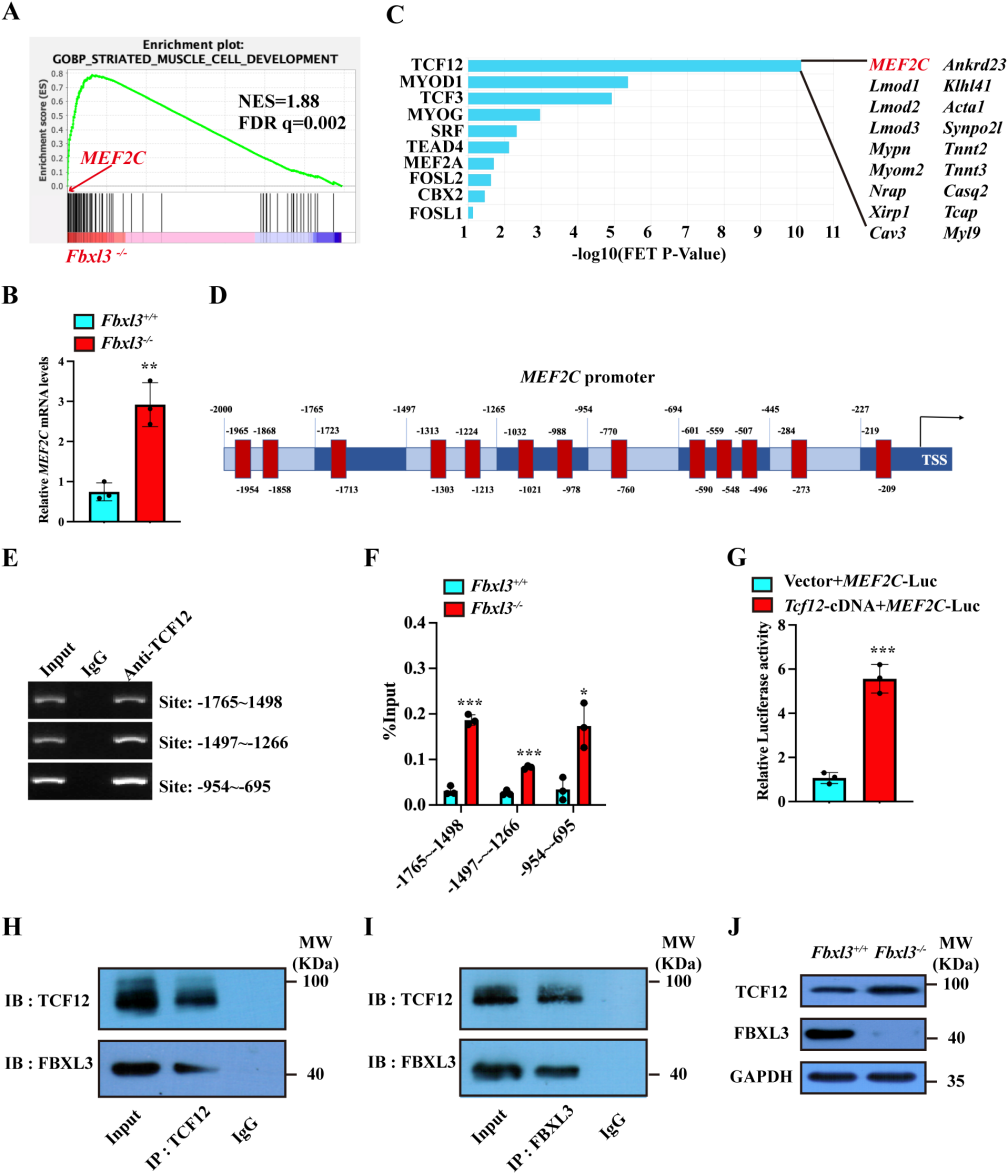
FBXL3 regulates the expression of MEF2C through the transcription factor TCF12. **(A)** RNA sequencing (RNA-Seq) was performed using *Fbxl3^+/+^* and *Fbxl3^-/-^* myoblasts. Gene set enrichment analysis shows the enrichment of genes involved in striated muscle cell development in *Fbxl3^-/-^* cells. **(B)** RT-qPCR analysis of the expression of *MEF2C* in *Fbxl3^+/+^* and *Fbxl3^-/-^* myoblasts (n=3). *P* values were determined by unpaired t test. **(C)** Identification of transcriptional factors involved in striated muscle cell development downstream of FBXL3. **(D)** The binding sites of TCF12 to MEF2C promoter (red) were predicted by the JASPAR database. **(E)** ChIP assay was performed using WT myoblasts to identify the binding sites of TCF12 on the MEF2C promoter. The numbers indicate the position of the consensus sequence upstream of the transcriptional start site of the MEF2C gene. IgG, immunoglobulin **(G)**. **(F)** RT-qPCR analysis of the percentage of input enrichment of TCF12 at specific sites on MEF2C promoter in *Fbxl3^+/+^* and *Fbxl3^-/-^* cells (n=3). *P* values were determined by unpaired t test. **(G)** Dual-luciferase reporter assay of MEF2C promoter activation by TCF12. 293T were co-transfected with 1000 ng empty vector or TCF12-cDNA, 1000 ng MEF2C-Luc, and 200 ng pRL-TK plasmid by Lipofectamine 2000 (n=3). *P* values were determined by unpaired t test with Welch’s correction. **(H, I)** In WT myoblasts, TCF12 and FBXL3 antibodies were used for immunoprecipitation, and Western blot was used to detect FBXL3 and TCF12. Normal IgG was used as the control. **(J)** Western blot was performed to detect TCF12 protein in the *Fbxl3^+/+^* and *Fbxl3^-/-^* cells. Values are mean ± SD. *P < 0.05, **P < 0.01, ***P < 0.001.

Furthermore, the result of the gene set enrichment analysis (GSEA) showed that FBXL3 deficiency resulted in the enrichment of the striated muscle cell development gene set (**Figure 5A**). These findings align with our observations that FBXL3 deletion enhances muscle repair. MEF2C emerged as enriched at the striated muscle cell development gene set (**Figure 5A**). Validation through RT-qPCR confirmed the upregulation of *MEF2C* (**Figure 5B**) in *Fbxl3^-/-^* cells.

By searching the ChEA3 database, we identified TCF12 as the candidate gene downstream of FBXL3 regulating MEF2C (muscle cell differentiation, **Figure 5C**). JASPAR prediction (http://jaspar.genereg.net/) revealed potential TCF12 binding sites on MEF2C promoter region (**Figure 5D**, **Supplementary Table S2**). CHIP assays covering 2000 bp upstream of the MEF2C promoter identified TCF12 enrichment in multiple consensus segments (**Figure 5E**). To investigate if FBXL3 regulates the binding of TCF12 to MEF2C promoter, we performed ChIP-qPCR using *Fbxl3^+/+^* and *Fbxl3^-/-^* myoblasts. The results revealed significantly increased TCF12 enrichment at the MEF2C promoter in *Fbxl3^-/-^* myoblasts compared to *Fbxl3^+/+^* myoblasts (**Figure 5F**). Additionally, RT-qPCR showed elevated *MEF2C* mRNA levels in WT primary myoblasts transduced with lentivirus-overexpressing TCF12 cDNA compared with WT primary myoblasts transduced with an empty vector (**Supplementary Figures S7A–C**). In contrast, the overexpression of FBXL3 in C2C12 myoblasts inhibited the expression of *MEF2C* (**Supplementary Figure S7D**).

For an assessment of TCF12’s ability to activate MEF2C promoter, a dual-luciferase reporter assay was conducted. 293T transfected with the TCF12-overexpressing plasmid exhibited significantly higher luciferase activity compared to cells transfected with an empty vector (**Figure 5G**). Given that FBXL3 functions as an E3 ubiquitin ligase within the SCF complex, we hypothesized that FBXL3 might be involved in degrading TCF12. Immunoprecipitation confirmed the physical association between FBXL3 and TCF12, indicating mutual precipitation between FBXL3 and TCF12 antibodies (**Figures 5H, I**).

To investigate the role of E3 ligase activity in the upregulation of TCF12 in *Fbxl3^-/-^* cells, a Western Blot experiment was conducted using the *Fbxl3^+/+^* and *Fbxl3^-/-^* cells and TCF12 antibody. The results demonstrated a higher amount of TCF12 in *Fbxl3^-/-^* compared with *Fbxl3^+/+^* cells (**Figure 5J**), suggesting that the elevated levels of TCF12 in *Fbxl3^-/-^* cells might be due to reduced degradation. Furthermore, immunofluorescence staining of MyHC revealed enhanced myotube formation when TCF12 was overexpressed in myoblasts (**Supplementary Figures S7E, F**).

### Verification of TCF12 as a target for FBXL3 degradation

**Figure 6:**
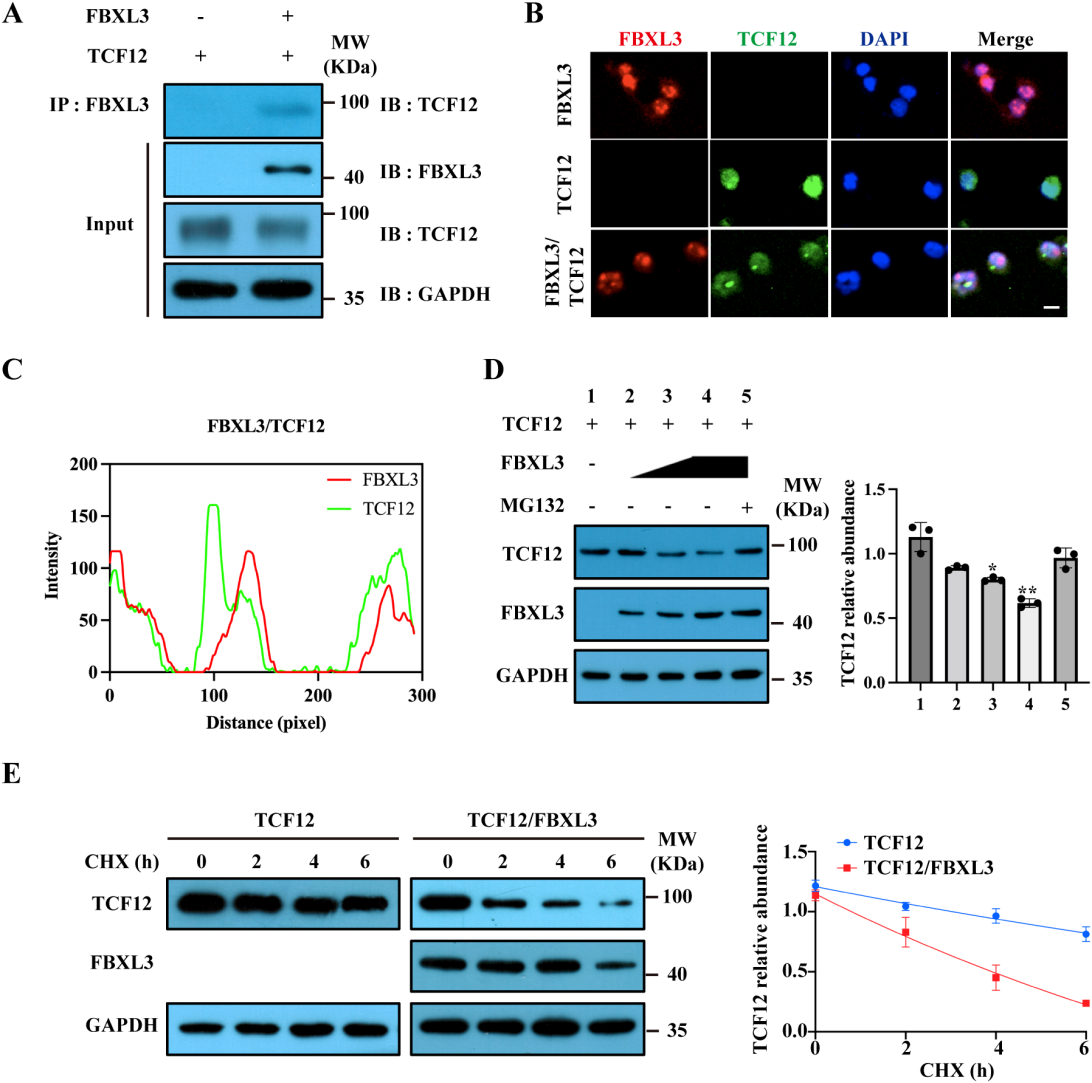
FBXL3 regulates TCF12 degradation. **(A)** Interaction between FBXL3 and TCF12. 293T cells were transfected with FBXL3 and TCF12, and immunoprecipitation was performed using anti-FBXL3 antibody. Western blot (IB) was used to detect TCF12. **(B)** Co-localization of TCF12 (green) and FBXL3 (red) in 293T cells transfected with indicated plasmids. Scale bars: 20 μm. **(C)** Fluorescence co-localization analysis according to (6B lower panel) was performed by ImageJ software. **(D)** FBXL3 inhibits TCF12 expression in a dose-dependent manner. Western blot analysis of TCF12 expression in 293T cells (left) and densitometric analysis of the relative levels of TCF12 normalized to GAPDH signals from three independent experiments (right). 293T cells were co-transfected with TCF12 with increasing amounts of FBXL3 in the presence or absence of MG132. The *P* values of group 1 vs 2 and 1 vs 3 were determined by unpaired t test with Welch’s correction. The *P* values of group 1 vs 4 and 1 vs 5 were determined by unpaired t test. **(E)** TCF12 turnover was accelerated in the presence of FBXL3. 293T cells were co-transfected with the indicated plasmids before treatment with 100 μg/ml CHX. Western blot was performed to determine TCF12 and FBXL3 using corresponding antibodies. Right panel: quantification of the effect of FBXL3 on TCF12 stability (n=3). Half-life was calculated using nonlinear, one-phase decay analysis by GraphPad Prism 8.0 (TCF12, 15.5 h, TCF12/FBXL3, 9.5 h; the half-life parameter, K, is significantly different when FBXL3 is co-expressed: *P < 0.05; **P < 0.01).

To biochemically confirm TCF12 as a binding partner for FBXL3, we co-expressed TCF12 and FBXL3 in 293T cells. Immunoprecipitation (IP) demonstrated an interaction between FBXL3 and TCF12 (**Figure 6A**). Immunofluorescence staining in 293T cells further confirmed the co-localization of TCF12 and FBXL3 (**Figures 6B, C**).

We subsequently investigated the impact of FBXL3 overexpression on TCF12 protein stability in 293T cells. FBXL3 overexpression dose-dependently reduced TCF12 protein levels, an effect counteracted by the 26S proteasome inhibitor MG132 (**Figure 6D**). This suggests that FBXL3 functions as an E3 ligase, orchestrating the degradation of TCF12 through the proteasome. To assess TCF12 turnover quantitatively, we conducted a cycloheximide (CHX) chase assay. The half-life of TCF12 protein decreased significantly from 15.5 h to 9.5 h when co-expressed with FBXL3 (**Figure 6E**).

### Loss of *Fbxl3* enhances muscle repair after IR injury in *Pax7Cre-ERT2* mice

The Pax7-Cre mice are constitutively active which may affect the function of satellite cells during embryonic/post-natal development. To separate a developmental role for *Fbxl3* (increased satellite cells) versus *Fbxl3* contributions to satellite cell regulation during regeneration, we generated a mouse model with satellite cell-specific FBXL3 knockout using the tamoxifen-inducible Pax7-CreER recombination system (**Supplementary Figure S8A**). The genotype of mouse was confirmed using PCR (**Supplementary Figure S8B**). *Fbxl3^fl/fl-Pax7cre-ERT2^* mice were treated with tamoxifen to delete *Fbxl3*, followed by IR injury and the TA muscle was harvested 7 days later (**Supplementary Figure S8C**). H&E staining demonstrated that compared to littermate *Fbxl3^fl/fl^* mice, the TA muscle of *Fbxl3^fl/fl-Pax7cre-ERT2^* mice exhibited higher numbers of CNF and a greater CSA of CNF after IR injury (**Supplementary Figures S8D–G**). Additionally, the expression of the eMyHC was higher in the TA muscle of *Fbxl3^fl/fl-Pax7cre-ERT2^* mice than *Fbxl3^fl/fl^* mice on day seven after IR injury (**Supplementary Figures S8H–J**). These results indicate that the absence of FBXL3 in satellite cells is beneficial for the regeneration of injured muscle fibers after excluding developmental effects.

## Discussion

Satellite cells, crucial for muscle repair, become activated undergo myogenic differentiation to generate new muscle fibers following muscle injury. The orchestrated involvement of MyoD, myogenin, Myf5, and Mrf4 is essential for effective myogenic regeneration (26). MyoD and Myf5 play pivotal roles in the initial proliferation phase of myoblasts (27, 28), while the collaborative action of MyoD and myogenin is indispensable for myogenic differentiation (29, 30). Our findings demonstrate the loss of FBXL3 amplifies myogenic regeneration in injured skeletal muscle by promoting the expression of MyoD, myogenin, and MEF2C. These insights contribute novel understanding to the regulatory mechanism governing muscle repair.

MRFs collaborate with myocyte enhancer factor 2 (MEF2) to facilitate the differentiation of myoblasts into myotubes (31). Initially identified as a myocyte-specific enhancer-binding factor, MEF2 recognizes the promoter of the muscle creatine kinase gene and becomes detectable within 2 hours after myoblasts are placed in mitogen-deficient medium (32). Vertebrates possess four MEF2 genes (MEF2A, MEF2B, MEF2C, and MEF2D), present in various cell types, including all three-types of muscle cells. MEF2C is the first expressed in skeletal muscle during embryogenesis (33). While MEF2 alone cannot induce myogenesis when transfected into fibroblasts, it can enhance myogenic differentiation when coexpressed with either MyoD or myogenin (34). The MEF2C promoter contains a skeletal muscle-specific control region capable of binding to both MyoD and MEF2. This suggests that the cooperative action of basic helix-loop-helix (bHLH) transcription factors and MEF2 factors is necessary to direct MEF2C expression (35). Satellite cells lacking MEF2A, C, and D proliferate normally in culture but exhibits an inability to differentiate (36). Homozygous null mutations of MEF2C leads to embryonic lethality due to abnormal heart development (37). Mice deficient in MEF2C specifically within the skeletal muscle are viable but display lower body weight compared to control mice (38). Despite being considered as a downstream target of the bHLH transcription factors like MyoD, myogenin, Myf5, and MRF4 (39, 40), MEF2 factors, including MEF2C, can reciprocally influence the transcription of *myogenin* (8) and *Mrf4* (41). In certain cell lines, myogenin, but not MyoD, induces MEF2C expression (42). Adiponectin regulates MEF2C during myogenic differentiation, establishing a positive feedback loop where MEF2C promotes adiponectin and AdipoR1 transcription, fostering myoblast differentiation (43). Additionally, MEF2C’s DNA binding activity is enhanced by phosphorylation induced by p38 in muscle cells (44, 45), and phosphorylation by skeletal myosin light chain kinase activates MEF2C to facilitate myogenic differentiation (46). Our findings revealed an elevated number of MyoD^+^ cells on single myofibers from *Fbxl3^scko^* mice compared to *Fbxl3^fl/fl^* mice, suggesting a potential role of MEF2C in MyoD expression regulation. Consequently, MEF2C and bHLH factors establish a positive feedback loop, amplifying myogenesis processes. Our study introduces FBXL3 as a novel regulator in myogenic differentiation by influencing the availability of MEF2C for myogenesis.

Earlier investigations demonstrated that FBXL21, an additional member of the F-box E3 ubiquitin ligase family, sharing a 84% amino acid sequence similarity with with FBXL3 (47), regulates myogenic differentiation through MYOZ1 ubiquitinaton (16). *Psttm* mice, carrying an *Fbxl21* hypomorph allele, manifested an excessive accumulation of MYOZ1 in the Z-line, resulting in sarcomere structure disruption, reduced muscle fiber diameter, and diminished grip strength (16). *Fbxl21* knockout in C2C12 cells impaired myogenic differentiation (16). Despite their sequence homology, FBXL21 and FBXL3 exert opposing effects on CRY stability (13, 14). Considering the functional antagonism between FBXL21 and FBXL3 in CRY degradation, our findings, indicating that FBXL3 deletion fosters myogenic differentiation and regeneration, align with this regulatory framework.

To elucidate how the loss of FBXL3 promotes satellite cell differentiation, we conducted RNA sequencing and gene set enrichment analysis (GSEA) using *Fbxl3^+/+^* and *Fbxl3^-/-^* primary myoblasts, and identified TCF12 as the candidate gene downstream of FBXL3 regulating MEF2C. TCF12, belonging to the basic helix loop helix E protein family, plays a pivotal role in the differentiation of diverse cell types (48, 49), such as muscle cells, hematopoietic stem cell (49), and neuron (50). It regulates lineage-specific gene expression by forming heterodimers with other bHLH E-proteins. Muscle-specific deletion of Tcf12 results in muscle loss and impedes the process of muscle regeneration (51). Previous studies demonstrated that TCF12 facilitates myogenic differentiation by binding to the E-box regions of MyoD and myogenin (7). Our findings suggest that FBXL3 plays a role in the degradation of TCF12. In the absence of FBXL3, elevated levels of TCF12 leads to increased expression of MEF2C. Consistently, ChIP and dual luciferase assays revealed that TCF12 can bind to the MEF2C promoter and driving its transcription.

While our study demonstrates the crucial role of FBXL3 in suppressing regenerative myogenesis and highlights its potential as a therapeutic target for enhancing muscle repair, several limitations should be acknowledged. Firstly, the study focuses on acute muscle injury models, and the long-term effects of FBXL3 modulation on muscle homeostasis and aging-related muscle decline, such as sarcopenia, remain to be explored. Understanding how FBXL3 influences muscle regeneration in the context of healthy aging could provide valuable insights into age-related muscle loss and potential interventions. Future studies should investigate the impact of FBXL3 modulation on muscle function and regeneration in aged animals and its interaction with other pathways implicated in sarcopenia, such as mitochondrial dysfunction and inflammation. Additionally, exploring the translational potential of targeting FBXL3 in human muscle disorders, including sarcopenia and muscular dystrophies, will be essential for advancing therapeutic strategies.

To recap, FBXL3 functions as a negative regulator of regenerative myogenesis by initiating the degradation of TCF12. The targeted deletion of FBXL3 in satellite cells enhances muscle regeneration, primarily through modulation of the TCF12/MEF2C signaling pathway.

## Data Availability

The original contributions presented in the study are included in the article/**Supplementary Material**. Further inquiries can be directed to the corresponding authors.
