# Supplementary material for: FBXL3 serves as a suppressor of regenerative myogenesis
Source: Front Immunol. 2025 Jul 18;16:1575712. doi: 10.3389/fimmu.2025.1575712 (PMC12313707; doi:10.3389/fimmu.2025.1575712)
Supplement: Supplementary file 1 [file DataSheet1.pdf]

Supplementary Material

FBXL3 serves as a suppressor of regenerative myogenesis

Wei He[a,b#], Shiyuan Han[a#], Yanming Wu[c#], Min Chen[c#], Ting Xue[a#], Hua You[c], Ying Chang[a], Song-Bai Liu[d], Yi Sun[e],Yinjiang Tang[e], Xinghong Shi[a], Xingyu Han[a], Zixin Ma[a], Panting Qian[a], Sha Geng[a], Chaofan Wu[a], Yating Liang[a], Yangxin Li[f], Yan Xu[g]*, Yao-Hua Song[a,c]*.

[a]Cyrus Tang Medical Institute, Collaborative Innovation Center of Hematology, State Key Laboratory of Radiation Medicine and Protection, Soochow University, Suzhou, 215123, People's Republic of China

[b]Department of Medical laboratory, the Second Affiliated Hospital of Hainan Medical University, Yehai road 368#, Longhua District, Haikou city, 570216, Hainan Province, P. R. China.

[c]Department of Cardiology, Suzhou Ninth People's Hospital, Suzhou Ninth Hospital Affiliated to Soochow University, 215200, Suzhou, P.R. China.

[d]Suzhou Key Laboratory of Medical Biotechnology, Suzhou Vocational Health College, Suzhou 215009, People's Republic of China

[e]Department of Cardiovascular Surgery, Fuwai Yunnan Cardiovascular Hospital, Kunming 650102, People's Republic of China

[f]Department of Cardiovascular Surgery of the First Affiliated Hospital & Institute for Cardiovascular Science, Soochow University, Suzhou Jiangsu 215000, People's Republic of China

[g]Department of General Medicine, The Second Xiangya Hospital, Central South University, Changsha, Hunan 410011, P.R. China

[#]These authors contributed equally to this work.

***Correspondence authors:**

Yao-Hua Song, M.D., Ph.D.

Cyrus Tang Hematology Center, Collaborative Innovation Center of Hematology,

Soochow University

199 Ren Ai Road, Suzhou 215123, China

Phone: 86-512-65880899/626,

Email: yaohua_song1@yahoo.com

Yan Xu, Ph.D

Department of General Medicine, The Second Xiangya Hospital of Central South University, 139 Middle Renmin Road, Changsha, Hunan 410011, P. R. China

Tel: +86-731-85295281; Fax: +86-731-85295281

E-mail: xuyan217@csu.edu.cn

This Additional file includes:

Figures S1 to S8

Table S1 to S2

Supplemental Figure 1

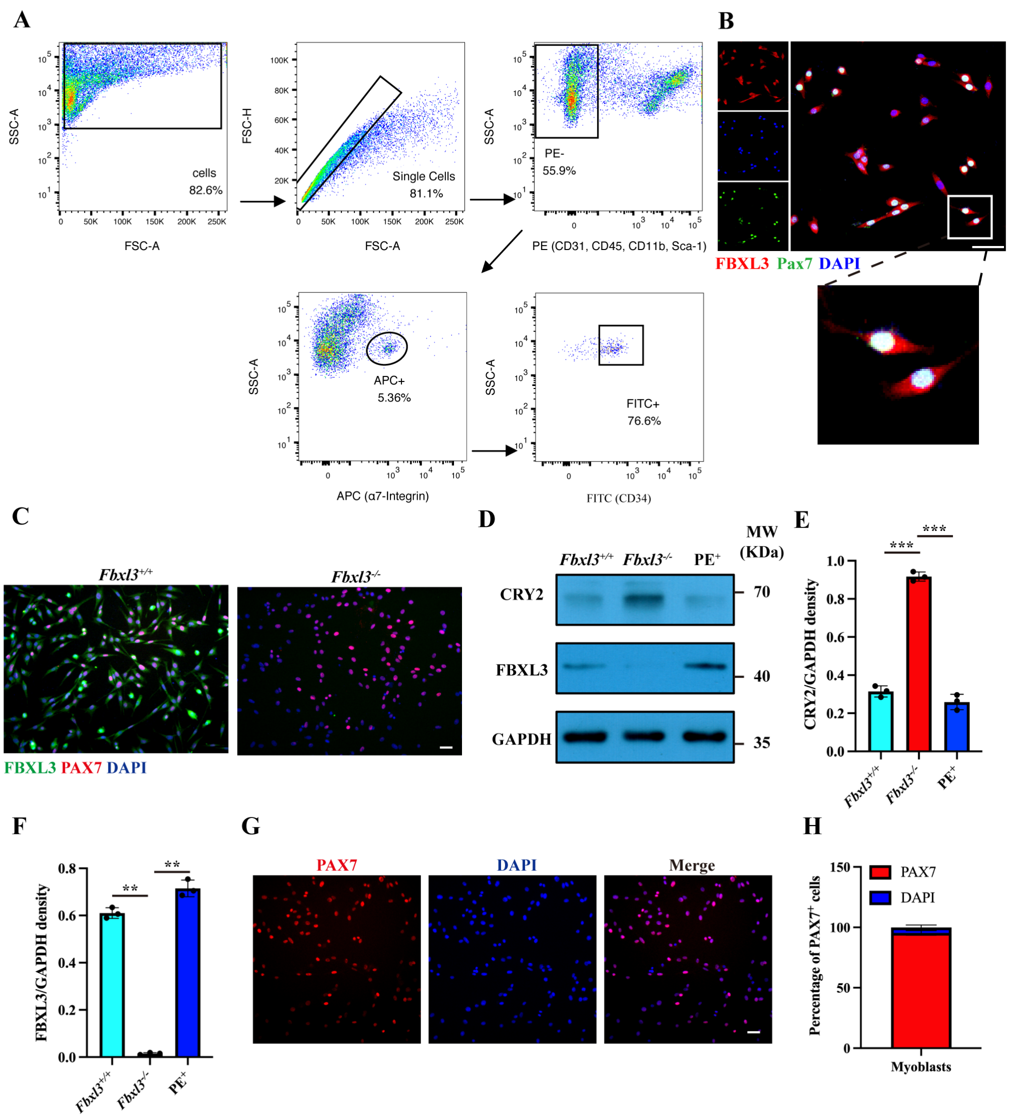

Supplementary Figure 1. Isolation of satellite cells by cell sorting, related to Figure 1. (A) Single-cell suspensions were obtained from hindlimb muscles. Satellite cells (CD31$^-$, CD45$^-$, CD11b$^-$, Sca-1$^-$, α7-Integrin$^+$, CD34$^+$) were sorted and used for analysis. (B) Freshly sorted primary myoblasts isolated from the TA muscle of C57BL/6J (WT) mice were immunostained with antibodies against FBXL3 (red) and PAX7 (green). The nuclei were stained with DAPI (blue). Scale bars: 50 μm. (C) Immunostaining of FBXL3 (green) and PAX7 (red) in *Fbxl3*$^{+/+}$ and *Fbxl3* $^{-/-}$ primary myoblasts cultured in growth medium. The nuclei were stained with DAPI (blue). Scale bar: 50 μm. (D-F) Western blot analysis of the protein level of CRY2, FBXL3 in *Fbxl3*$^{+/+}$, *Fbxl3*$^{-/-}$, and PE$^+$ lineage cells (n=3). *P* values were determined by one-way ANOVA with Dunnett's T3 multiple comparisons test. (G-H) Immunostaining of PAX7 (red) in primary myoblasts cultured in growth medium. The

nuclei were stained with DAPI (blue). Scale bar: 50 μm. Values are mean ± SD. **$P < 0.01$, ***$P < 0.001$.

Supplemental Figure 2

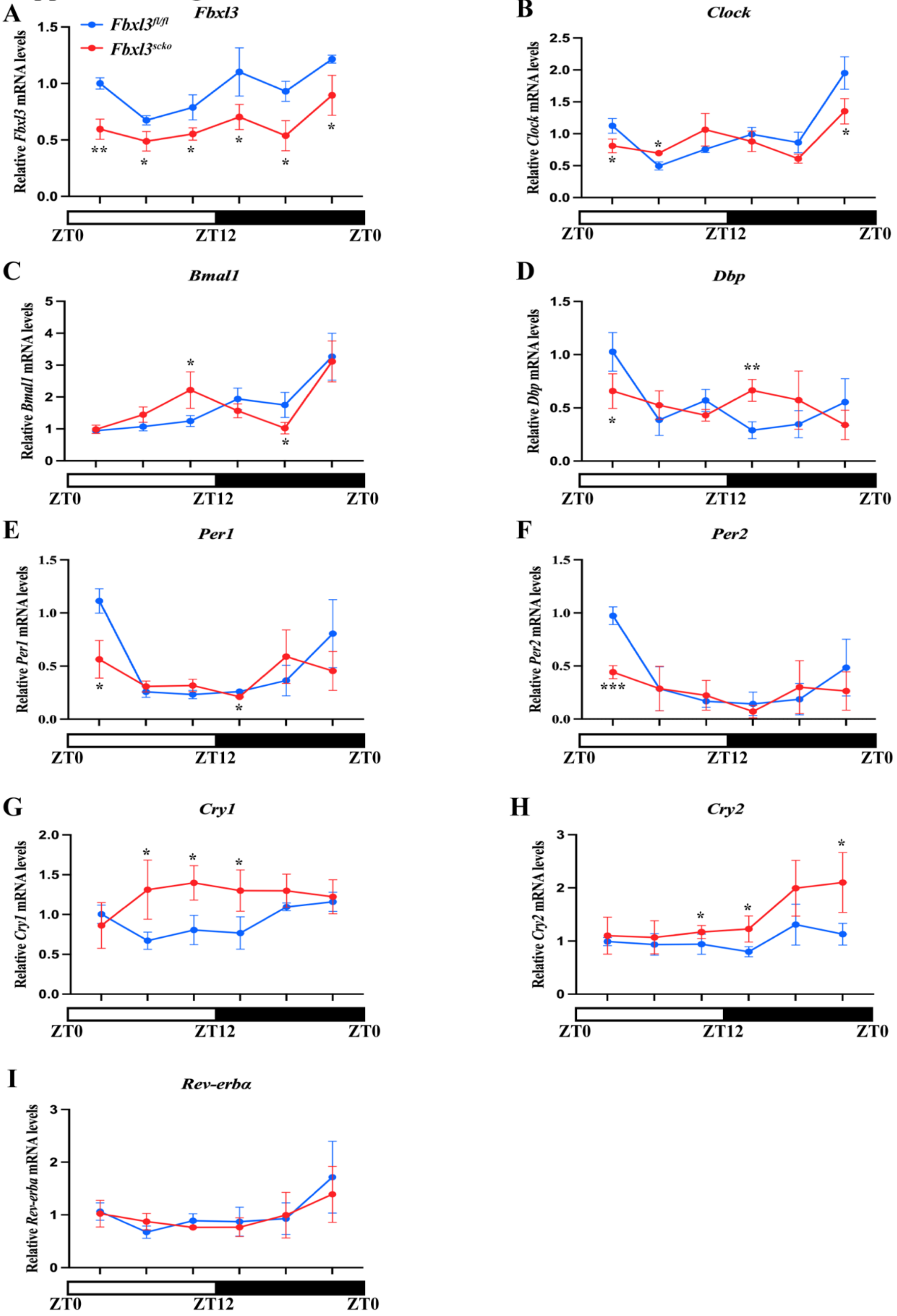

Supplementary Figure 2. FBXL3 deficiency affects the expression of circadian clock-related genes, related to Figure 1. (A) Temporal profiles of the expressions of *Fbxl3* mRNA in the TA

muscles from *Fbxl3*$^{fl/fl}$ and *Fbxl3*^{scko} mice. *Fbxl3*$^{fl/fl}$ mice expression values at ZT2 in the graph were defined as 1.0. The light was on between ZT0 and ZT12 and off between ZT12 and ZT0. n = 3 mice in each group at different time points. (B-I) Temporal profiles of the expressions of circadian clock gene mRNA in the TA muscles from *Fbxl3*$^{fl/fl}$ and *Fbxl3*^{scko} mice. n = 3 mice in each group at different time points. p values determined by unpaired t test. Values are mean ± SD. **P* < 0.05, ** *P* < 0.01, *** *P* < 0.001.

Supplemental Figure 3

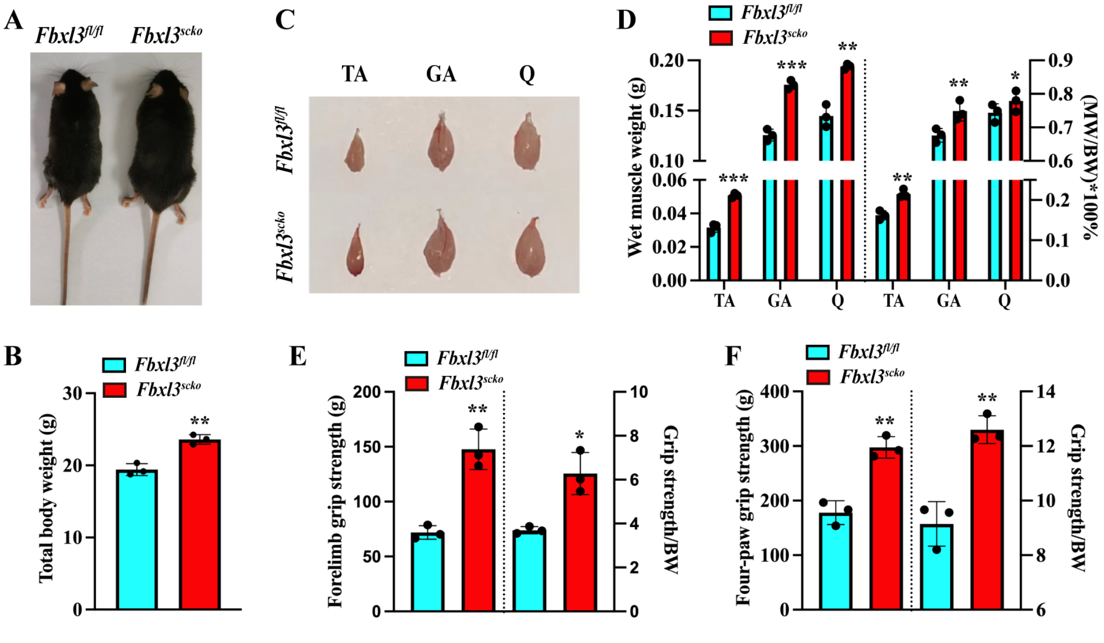

Supplementary Figure 3. Deletion of FBXL3 in satellite cells increases muscle weight and strength, related to Figure 2. (A) Representative photos of 8-week-old *Fbxl3$^{fl/fl}$* and *Fbxl3^{scko}* mice. (B) Average total body weight. (C) Representative images of tibialis anterior (TA), gastrocnemius (GA) and quadriceps (Q) muscles from *Fbxl3$^{fl/fl}$* and *Fbxl3^{scko}* mice. (D) Absolute and body weight-normalized wet weight of TA, gastrocnemius and quadriceps muscles. MW: Muscle Weight. BW: Body Weight. (E) Absolute and body weight-normalized forelimb grip strength. (F) Absolute and body weight-normalized four-limbs grip strength. n = 3 in each group. *P* values determined by unpaired t test. Values are mean ± SD. *$P < 0.05$, ** $P < 0.01$, *** $P < 0.001$.

Supplemental Figure 4

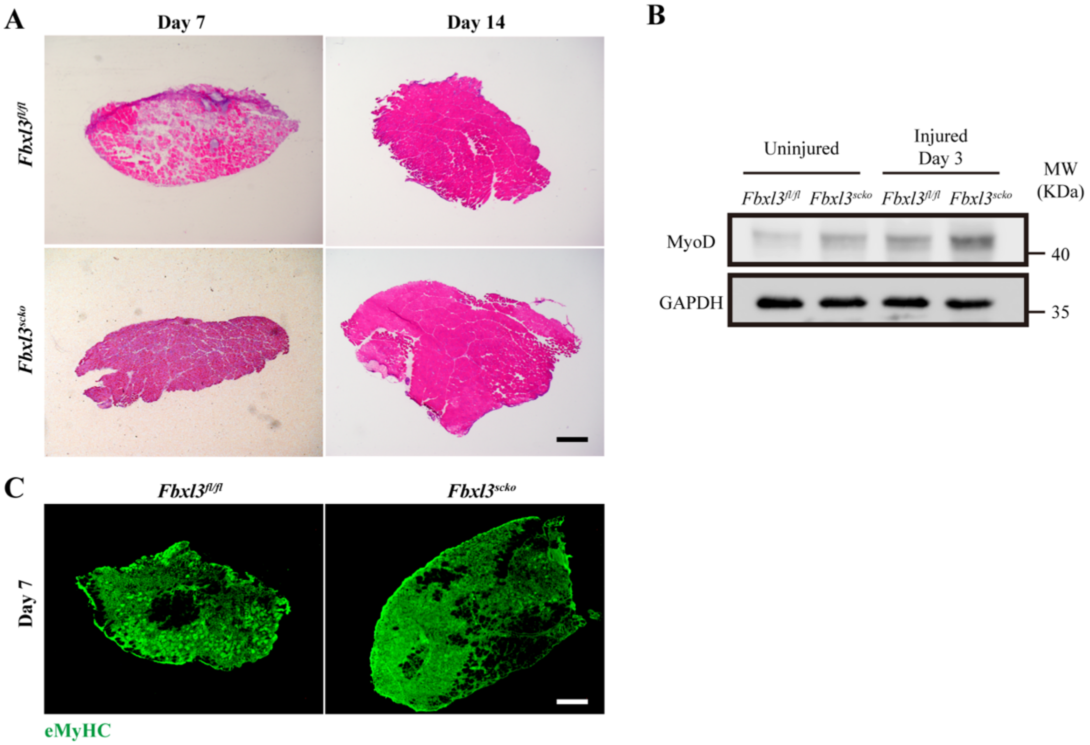

Supplementary Figure 4. Loss of FBXL3 promotes myogenesis after IR injury, related to Figures 2 and 3. (A) Full scan images of H&E-stained sections of the TA muscle from *Fbxl3*^{scko} and *Fbxl3*$^{fl/fl}$ littermates at indicated time points after IR injury, corresponding to Figure 2A. Scale bars: 500 µm. (B) Western blot analysis of relative protein levels of MyoD in uninjured and IR-injured TA muscles of *Fbxl3*^{scko} and *Fbxl3*$^{fl/fl}$ mice 3 days post-IR injury. (C) Full scan images of immunostaining of eMyHC (green) for regenerating myofibers in TA muscles from *Fbxl3*^{scko} and *Fbxl3*$^{fl/fl}$ mice 7 days after IR-induced injury, corresponding to Figure 3H. Scale bars: 500 µm.

Supplemental Figure 5

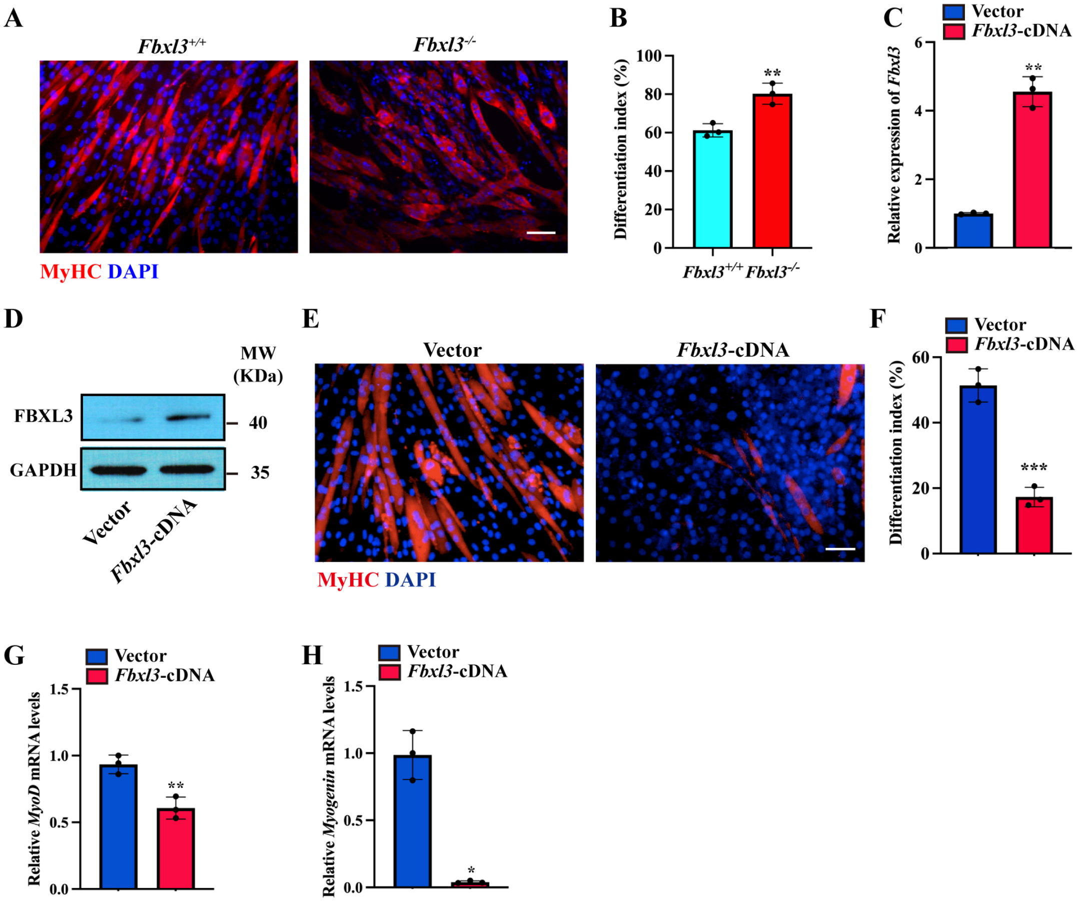

Supplementary Figure 5. FBXL3 is involved in the differentiation of myoblasts, related to Figure 3 and 4. (A) Immunostaining of MyHC (red) in *Fbxl3*$^{+/+}$ and *Fbxl3*$^{-/-}$ myoblasts. The cells were cultured in a differentiation medium for 6 days. Nuclei are counterstained with DAPI (blue). Scale bar: 100 µm. (B) Differentiation index (percentage of nuclei within MyHC^{+} myotubes) of *Fbxl3*$^{+/+}$ and *Fbxl3*$^{-/-}$ myoblasts. (C) RT-qPCR analysis of mRNA levels of *Fbxl3* (n=3, *P* values were determined by unpaired t test with Welch's correction) in C2C12 myoblasts transduced with lentivirus overexpressing FBXL3 cDNA or empty vector. (D) Western blot analysis of protein levels of FBXL3 in C2C12 myoblasts transduced with lentivirus overexpressing FBXL3 cDNA or empty vector. (E) Immunostaining of MyHC (red) in C2C12 transduced with lentivirus expressing empty vector or FBXL3-cDNA. These cells were cultured in a differentiation medium for 6 days. (F) Differentiation index (percentage of nuclei within MyHC^{+} myotubes) of C2C12-Lv5 and C2C12-FBXL3 overexpressing cells cultured in differentiation medium for 6 days. (G-H) RT-qPCR analysis of mRNA levels of *MyoD* (n=3, p values were determined by unpaired t test) and *Myogenin* (n=3, p values were determined by unpaired t test with Welch's correction) in C2C12 myoblasts transduced

with lentivirus overexpressing FBXL3 cDNA or empty vector. n = 3 in each group. Values are mean ± SD. $*P < 0.05$, $**P < 0.01$, $***P < 0.01$.

Supplemental Figure 6

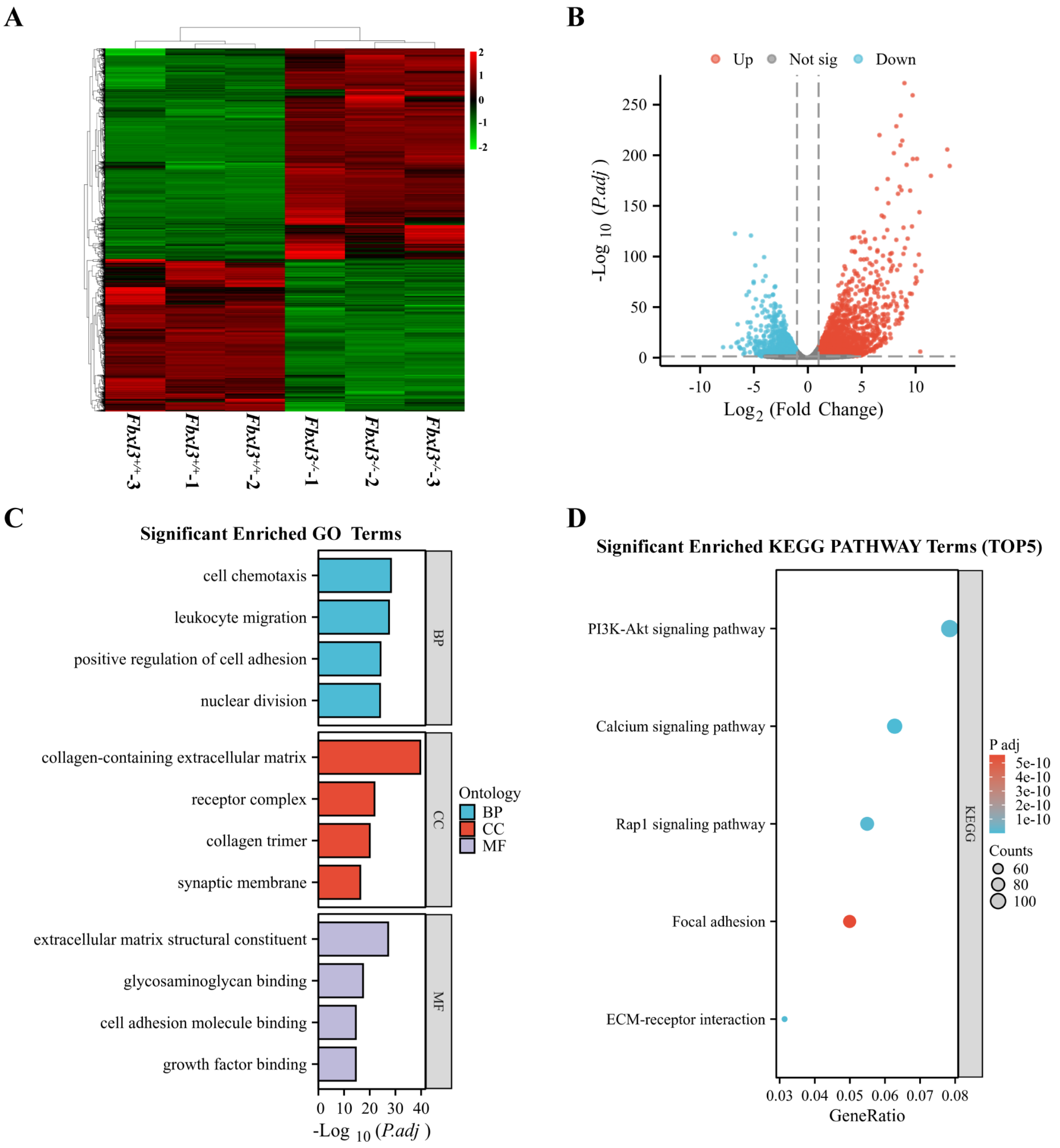

Supplementary Figure 6. Bioinformatics analysis of RNA sequencing results of *Fbxl3*$^{+/+}$ and *Fbxl3*$^{-/-}$ primary myoblasts, related to Figure 5. (A)The heatmap of differential expression gene. (B) The volcano map of differential expression gene. The blue dots represent down-regulated differentially expressed genes, the red dots represent up-regulated differentially expressed genes, and the gray dots represent no differentially expressed genes. (C) GO analysis of differentially expressed genes. BP: Biological process, CC: Cellular component, MF: Molecular function. (D) KEGG analysis of differentially expressed genes.

Supplemental Figure 7

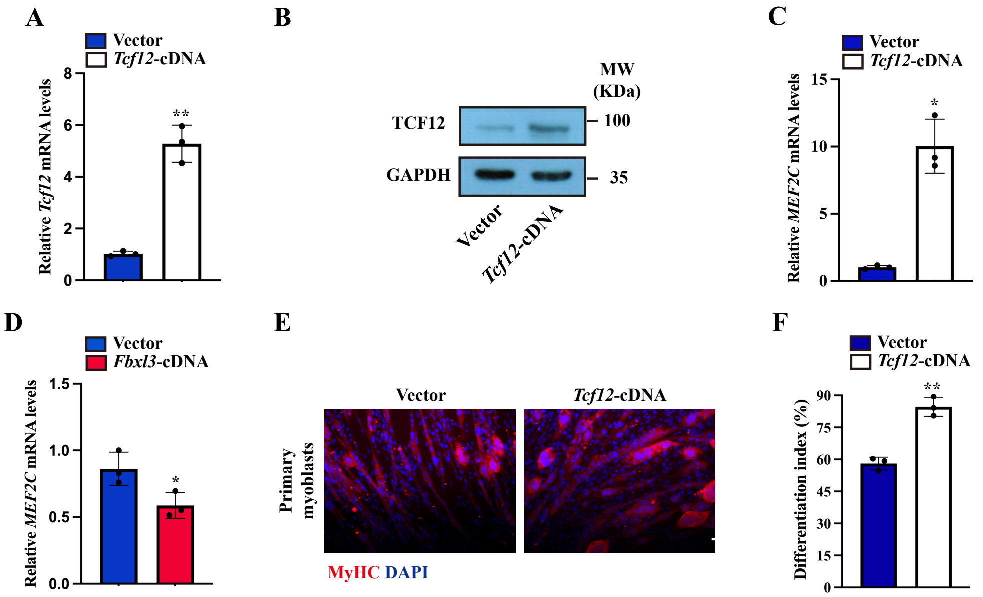

Supplementary Figure 7. Transcription factor TCF12 promotes the expression of MEF2C and myogenic differentiation, related to Figure 5. (A) RT-qPCR analysis of mRNA levels of *Tcf12* (n=3, p values were determined by unpaired t test with Welch's correction) in primary myoblasts transduced with lentivirus overexpressing TCF12 cDNA or empty vector. (B) Western blot analysis of protein levels of TCF12 in primary myoblasts transduced with lentivirus overexpressing TCF12 cDNA or empty vector. (C) RT-qPCR analysis of mRNA levels of *MEF2C* (n=3, p values were determined by unpaired t test with Welch's correction) in primary myoblasts transduced with lentivirus overexpressing TCF12 cDNA or empty vector. (D) RT-qPCR analysis of mRNA levels of *MEF2C* (n=3, p values were determined by unpaired t test) in C2C12 myoblasts transduced with lentivirus overexpressing FBXL3 cDNA or empty vector. (E) Immunostaining of MyHC (red) in myoblasts transduced with lentivirus expressing empty vector or TCF12-cDNA cultured in differentiation medium for 6 days. Nuclei are counterstained with DAPI (blue). Scale bar: 100 µm. (F) Differentiation index (percentage of nuclei within MyHC$^+$ myotubes) of primary myoblasts transfected with lentivirus carrying either empty Lv5 vector or TCF12 cDNA cultured in differentiation medium for 6 days. n = 3 independent experiments. *P* values were determined by unpaired t test. Values are mean ± SD. *$P < 0.05$, **$P < 0.01$.

Supplemental Figure 8

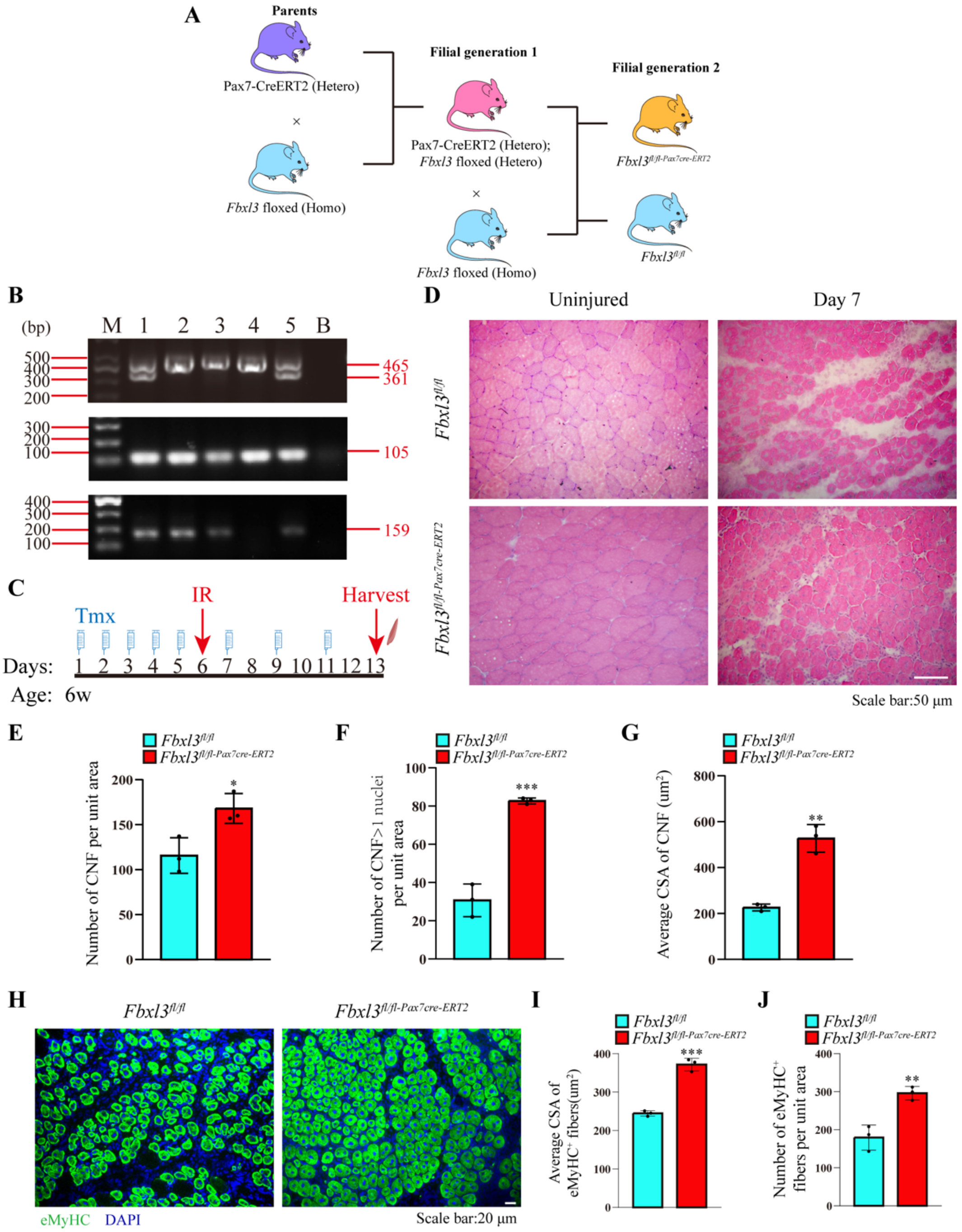

Supplementary Figure 8. Loss of FBXL3 enhances muscle repair after IR injury in *Pax7Cre-ERT2* mice, related to Figure 2 and 3. (A) Breeding strategy for obtaining *Fbxl3*$^{fl/fl\text{-}Pax7cre\text{-}ERT2}$ and littermate *Fbxl3*$^{fl/fl}$ mice. (B) The genotype of *Fbxl3*$^{fl/fl\text{-}Pax7cre\text{-}ERT2}$ and littermate *Fbxl3*$^{fl/fl}$ mice were identified by PCR and agarose gel electrophoresis using genomic DNA. FBXL3-floxed (Homo) mice genotype PCR product is 465 bp. For the Pax7-CreERT2 mice, wild type genotype PCR product is 105 bp, mutant PCR product is 159 bp, and heterozygote PCR product is 105 and 159 bp. Mouse #2,

3 had 465 bp, 105 bp and 159 bp bands; therefore, these mice are *Fbxl3*$^{fl/fl\text{-}Pax7cre\text{-}ERT2}$. Mouse #4 had 465 and 105 band; therefore, these mice are FBXL3-floxed (Homo). M: marker, B: blank, no template DNA control. (C) Experimental scheme showing mice injected with tamoxifen (Tmx) for 5 days, then with IR injury on day 6, followed by 3 more days of Tmx injections. Finally, the muscles were harvested on the seventh day post-injury. (D) H&E-stained sections of the TA muscle from *Fbxl3*$^{fl/fl\text{-}Pax7cre\text{-}ERT2}$ and *Fbxl3*$^{fl/fl}$ littermates at indicated time points after IR injury. Scale bars: 50 μm. (E-G) Quantification of myogenic regeneration. Numbers of centronucleated fibers (CNF) per field (0.1 mm^2) (E), the number of myofibers containing more than one central nucleus (F) and average CSA of regenerating myofibers (G) at 7 days after IR injury. (H-J) Immunostaining of eMyHC (green), quantification of average cross-sectional area (CSA) of eMyHC$^+$ fibers, and the number of eMyHC$^+$ fibers per field (0.08 mm^2) in TA muscles from *Fbxl3*$^{fl/fl\text{-}Pax7cre\text{-}ERT2}$ and *Fbxl3*$^{fl/fl}$ mice 7 days after IR-induced injury. Scale bars: 20 μm. n = 3 independent experiments. *P* values were determined by unpaired t test. Values are mean ± SD. $*P < 0.05$, $**P < 0.01$, $***P < 0.001$.

Supplemental Table 1: Sequences of qPCR or PCR primers.

Gene	Forward	Reverse
Fbxl3	CCTGACTTGTGGCGATGTTTT	ACTGTAGGTGGTTTGAGTGCC
Pax7	CAGTGTGCCATCTACCCATGCTTA	GGTGCTTGGTTCAAATTGAGCC
MyoD	TGGGATATGGAGCTTCTATCGC	GGTGAGTCGAAACACGGATCAT
Myogenin	CCATCCAGTACATTGAGCGCCT	CTGTGGGAGTTGCATTCACTGG
Tcf12	ATGTACTGTGCTTATCCTGTCCC	GGTGCATATACCGTTTTCCCATT
MEF2C	ATCCCGATGCAGACGATTCAG	AACAGCACACAATCTTTGCCT
Gapdh	CATCACTGCCACCCAGAAGACTG	ATGCCAGTGAGCTTCCCGTTCAG
Clock	GGCTGAAAGACGGCGAGAACTT	GTGCTTCCTTGAGACTCACTGTG
Bmal1	ACCTCGCAGAATGTCACAGGCA	CTGAACCATCGACTTCGTAGCG
Per1	GAAACCTCTGGCTGTTCCTACC	AGGCTGAAGAGGCAGTGTAGGA
Per2	CTGCTTGTTCCAGGCTGTGGAT	CTTCTTGTGGATGGCGAGCATC
Cry1	GGTTGCCTGTTTCCTGACTCGT	GACAGCCACATCCAACTTCCAG
Cry2	GGACAAGCACTTGGAACGGAAG	ACAAGTCCCACAGGCGGTAGTA
Rev-erb	GAAGAGTGACCGCACAGATTG	TCCGAAAGAAACCCTTACAGC
Dbp	ACACCGCTTCTCAGAGGAGGAA	TCTCGACCTCTTGGCTGCTTCA

MEF2C promoter -2000~-1766	CTTATTGTTTGGGAATTAAA	TTCTCTCCATTACTTCTTC
MEF2C promoter -1765~-1498	GAAGAAGTAATGGAGAGAA	ATAAGTGCCATTACCACAA
MEF2C promoter -1497~-1266	TTGTGGTAATGGCACTTAT	TCAAACTTCTACATCACACA
MEF2C promoter -1265~-955	TGTGTGATGTAGAAGTTTGA	ATAGACCTGAATGGCATCA
MEF2C promoter -954~-695	TGATGCCATTCAGGTCTAT	TAAGAAGGAGTATGCTAGGAC
MEF2C promoter -694~-446	GTCCTAGCATACTCCTTCTTA	ACCCACAATGCTACTGTAC
MEF2C promoter -445~-228	GTACAGTAGCATTGTGGGT	CACACACTTGCTTCATTTCA
MEF2C promoter -227~-1	TGAAATGAAGCAAGTGTGTG	TCTACACTGTTAGTTCCAAT
Fbxl3$^{fl/fl}$ identification	CCAACATCGCTGTCCTCCAC	GCCTGGTAGTCCTGGGTTCT
Pax7-Cre-WT identification	CTCCTCCACATTCCTTGCTC	CGGCCTTCTTCTAGGTTCTG

Pax7-Cre-Mut identification	GCGGTCTGGCAGTAAAAACTATC	GTGAAACAGCATTGCTGTCACTT
Pax7Cre-ERT2-WT identification	AGACTCAGGGCTTGGGAAGG	CTGTGCTGGGACTTCTTCCT
Pax7Cre-ERT2-Mut identification	AAAGACGGCAATATGGTGGA	CTGTGCTGGGACTTCTTCCT
Fbxl3 overexpression	ACATGCATGCATGTGCCACCATGTACCCATACGACGTCCCAG	CGCGGATCCGCGTTACCAAGTAGGCATCATGTCTG
Tcf12 overexpression	ACATGCATGCGCCACCATGTACCCATACGACGTCCCAGACTACGCTAATCCCCAGCAGCAGCGCATG	ATAGTTTAGCGGCCGCTTACAGATGACCCATAGGGTTGG

Supplemental Table 2: The putative consensus sequences for TCF12 in the *MEF2C* promoter predicted by JASPAR database.

Sequence ID	Start	End	Predicted sequence
MEF2C	-1965	-1954	TCACAGCTCCTA
MEF2C	-1868	-1858	GACAGAGGAAG
MEF2C	-1723	-1713	GGCAGAAGGGC
MEF2C	-1313	-1303	AACTGCTCCTG
MEF2C	-1224	-1213	GAGTAGCTGTTT
MEF2C	-1032	-1021	ACTCAGCTAGCT
MEF2C	-988	-978	GAGAGGTGCTT
MEF2C	-770	-760	AACAGGTACTT
MEF2C	-601	-590	TTCCAACTGAAT
MEF2C	-559	-548	ATTCACCTGCTA
MEF2C	-507	-496	TACCAGCTAAAT
MEF2C	-284	-273	AATGAGCTGCGG
MEF2C	-219	-209	AGCAAGTGTGT

Note: The highlighted part is the experimentally confirmed binding consensus sequences for TCF12 in the *MEF2C* promoter.
